# P2RX7 promotes osteosarcoma progression and glucose metabolism by enhancing c-Myc stabilization

**DOI:** 10.1186/s12967-023-03985-z

**Published:** 2023-02-20

**Authors:** Gaohong Sheng, Yuan Gao, Qing Ding, Ruizhuo Zhang, Tianqi Wang, Shaoze Jing, Hongqi Zhao, Tian Ma, Hua Wu, Yong Yang

**Affiliations:** 1 Department of Orthopedics, Tongji Hospital, Tongji Medical College, Huazhong University of Science and Technology, Jiefang Avenue 1095, Wuhan, 430030 China; 2 Department of Oncology, Tongji Hospital, Tongji Medical College, Huazhong University of Science and Technology, Wuhan, 430030 China; 3 Shanxi Bethune Hospital, Tongji Shanxi Hospital, Shanxi Academy of Medical Sciences, Third Hospital of Shanxi Medical University, Taiyuan, 030032 China

**Keywords:** Glucose metabolism, Metabolic reprogramming, P2RX7, c-Myc, Osteosarcoma

## Abstract

**Background:**

Osteosarcoma is the most common malignant tumor in bone and its prognosis has reached a plateau in the past few decades. Recently, metabolic reprogramming has attracted increasing attention in the field of cancer research. In our previous study, P2RX7 has been identified as an oncogene in osteosarcoma. However, whether and how P2RX7 promotes osteosarcoma growth and metastasis through metabolic reprogramming remains unexplored.

**Methods:**

We used CRISPR/Cas9 genome editing technology to establish P2RX7 knockout cell lines. Transcriptomics and metabolomics were performed to explore metabolic reprogramming in osteosarcoma. RT-PCR, western blot and immunofluorescence analyses were used to determine gene expression related to glucose metabolism. Cell cycle and apoptosis were examined by flowcytometry. The capacity of glycolysis and oxidative phosphorylation were assessed by seahorse experiments. PET/CT was carried out to assess glucose uptake in vivo.

**Results:**

We demonstrated that P2RX7 significantly promotes glucose metabolism in osteosarcoma via upregulating the expression of genes related to glucose metabolism. Inhibition of glucose metabolism largely abolishes the ability of P2RX7 to promote osteosarcoma progression. Mechanistically, P2RX7 enhances c-Myc stabilization by facilitating nuclear retention and reducing ubiquitination-dependent degradation. Furthermore, P2RX7 promotes osteosarcoma growth and metastasis through metabolic reprogramming in a predominantly c-Myc-dependent manner.

**Conclusions:**

P2RX7 plays a key role in metabolic reprogramming and osteosarcoma progression via increasing c-Myc stability. These findings provide new evidence that P2RX7 might be a potential diagnostic and/or therapeutic target for osteosarcoma. Novel therapeutic strategies targeting metabolic reprogramming appear to hold promise for a breakthrough in the treatment of osteosarcoma.

**Supplementary Information:**

The online version contains supplementary material available at 10.1186/s12967-023-03985-z.

## Background

Osteosarcoma (OS) is the most common primary malignant tumor of bone with an incidence of 1–3/million/year worldwide. The first peak of incidence arises in children and adolescents and a second minor peak in those aged > 50 years [1]. OS is characterized by the presence of transformed osteoblasts producing osteoid and immature bone. There are several histological types in OS with distinct biological and molecular behaviors, suggesting a high degree of heterogeneity [2]. OS pathogenesis has not been clearly elucidated, however, many environmental and genetic risk factors have been shown to be associated with osteosarcomagenesis and progression, such as age, radiation, bone diseases, genomic instability and so on [3]. OS most frequently occurs in the metaphysis of long extremity bones, including femur, tibia and humerus, while less commonly in axial skeleton, skull, jaw or pelvis. OS exhibits a very strong propensity for local invasion, distant metastasis, most commonly in the lungs, and recurrence. Since the introduction of chemotherapy in the early 1970s, the 5-year survival rate has reached approximately 70% for non-metastatic disease [4]. However, among patients with metastasis or recurrence, the overall survival rate remains dismal at around 20%. Moreover, survival rate has not been improved virtually over the past 4 decades [5, 6]. Therefore, more basic research and clinical trials are urgently needed to break this dilemma [7, 8].

In the past two decades, increasing attention has been drawn to the issue of metabolic reprogramming in cancer. In general, glucose metabolism reprogramming is common and required to fulfil the increase in biosynthesis during tumor development and progression. Surprisingly, high rate of glucose metabolism has only recently been considered as a hallmark of cancer biology, although Warburg effect (also known as aerobic glycolysis) was first described by Otto Warburg in the 1920s [9, 10]. Such renewed recognition might be due to the advances in our understanding of mechanisms by which certain oncogenic drivers affect the pattern and level of glucose metabolism in cancer [11]. For instance, transcription factor sine oculis homeobox 1 (SIX1) can facilitate tumor growth and metastasis by enhancing the Warburg effect [12]. Furthermore, the long-standing view that mitochondrial metabolism is inconsequential for tumor growth has been challenged. Instead, recent evidences both in humans and mice indicate that mitochondrial metabolism is also required and active in multiple cancers [13–15].

Purinergic receptors are composed of two families, including metabotropic P1 receptors and P2 family, which can be further divided into metabotropic P2Y and ionotropic P2X subfamilies. As a member of P2X subfamily, P2X7 receptor (P2RX7) is a ligand-gated cation-selective channel with two transmembrane domains [16]. Under physiological conditions, P2RX7 will be activated by the agonist ATP. Recently, P2X7R has received increasing attention since its unique structure and versatile functions [17]. Emerging evidences have highlighted that widely expressed P2RX7 is closely associated with a variety of physiological and pathological processes, including inflammation, immune response, pain, neuronal disorder, as well as cancer. Upregulation and activation of P2RX7 exhibit a pro-tumoral effect in various malignant tumors, such as leukemia [18], lung cancer [19], breast cancer [20], skin cancer [21], neuroblastoma [22], pancreatic ductal adenocarcinoma [23] and esophageal squamous cell carcinoma [24]. Nevertheless, there are also controversial reports that point out the opposite effects of P2RX7, inhibiting tumor development and progression [25–27]. Further research is needed to clarify the actual role of P2RX7 in tumorigenesis, depending on cancer types, activation pattern, splice variants [28] and tumor microenvironment (TME), such as acidosis, hypoxia and high extracellular ATP (eATP) concentration [29, 30]. Interestingly, P2RX7 is considered to be a critical modulator in cancer metabolic reprogramming [31]. Activation of P2RX7 enables HEK293 cells to fulfil increased energy demands for tumor growth through promoting both glycolysis and oxidative respiration via upregulation of the expression of numerous genes related to glucose metabolism [32, 33]. In leukemia cells, P2RX7 inhibition markedly impairs energy metabolism, thereby suppressing cancer cell proliferation and invasion [34]. Function loss of P2RX7 can also disrupt systematic energy homeostasis in mice [35]. With regard to OS, studies have also been conducted on the role and potential mechanisms of glucose metabolism in tumor progression [36–38]. Previous studies have suggested that P2RX7 is involved in biological behavior of OS cells [39, 40]. However, to our knowledge, it remains unclear whether and how P2RX7 regulates OS growth and metastasis through metabolic reprogramming.

As a transcriptional factor, c-Myc is overexpressed in many cancers and its oncogenic role has been extensively investigated [41, 42]. Notably, c-Myc participates in multiple metabolism-related pathways, such as glycolysis [43], mitochondrial respiration [44] and glutaminolysis[45, 46], via regulating the expression of metabolic modulators. Therefore, c-Myc can promote energy production and biosynthesis to help tumor growth, metastasis and survival under adverse conditions. It is noteworthy that recent articles have suggested the pivotal role of c-Myc in metabolic reprogramming of OS [47, 48].

In this study, we first demonstrated that P2RX7 promotes tumor growth and metastasis through metabolic reprogramming. Then we investigated the role of P2RX7 in regulating the expression of metabolism-related genes. Furthermore, P2RX7 enhances c-Myc stabilization through increasing nuclear location and reducing ubiquitin-dependent degradation, thus reshaping glucose metabolism and contributing OS progression. Our results provide new evidence for the significance of metabolic reprogramming in OS and reveal a potential mechanism by which P2RX7 enhances glucose metabolism and facilitates OS development through increasing c-Myc stabilization. These findings suggest that targeting P2RX7-mediated metabolic reprogramming might be promising to develop more effective therapeutic strategies for OS treatment.

## Methods

### Cell culture and treatment

OS cell lines (MNNG/HOS and U-2 OS) were purchased from the Cell Bank of Chinese Academy of Sciences (Shanghai, China) and routinely cultured in Dulbecco’s modified Eagle’s medium F12 (DMEM/F12; Gibco, USA) containing 10% fetal bovine serum (FBS; Gibco, USA) and 1% penicillin/streptomycin (Sigma-Aldrich, USA) in a humidified incubator with 5% CO_2_ at 37 °C. The medium was refreshed every 2 or 3 days. When cells reached about 80% confluence, they were passaged for subsequent experiments or frozen. In terms of specific conditions like serum starvation and hypoxia, cells were maintained in medium supplemented with 2% FBS under 1% O_2_. Cells were treated with 2-DG (2.5 mM) and oligomycin (100 μM) for indicated times. Proteasome inhibitor MG132 (10 μM) was added to cultured cells for 2 h.

### Construction of cell lines

P2RX7 knockout cells were constructed by CRISPR/Cas9 genome editing technology. The single guide RNA (sgRNA) targeting human P2RX7 was cloned into pLentiCRISPR v2 (Plasmid #52961, Addgene) and the synthesized plasmids were then transfected into 293 T cells for lentivirus production. After lentivirus infection and puromycin resistance screening, stably transfected cell lines were obtained. Then, single cell suspensions obtained after sufficient dilution were planted and proliferated in a 96-well plate. The knockout clones were identified by genomic DNA extraction, designated region amplification, sequencing and comparison with nuclease target sites. Five P2RX7 knockout clones of MNNG/HOS cell line and 9 clones of U-2 OS cell line were mixed for further experiments, respectively. The same number of clones infected with control sgRNA were mixed and used as a control. The genomic sequences targeted by sgRNA#1 and sgRNA#2 in human P2RX7 are 5′-GGCTGACAGCACTTGCACCA-3′ and 5′-TTCTGTGCACACCAAGGTGA-3′, respectively.

In terms of generation of P2RX7 overexpression cell lines, the lentiviral vector (Ubi-MCS-3FLAG-SV40-Puro) encoding P2RX7 and empty control vector was purchased from Genechem (Shanghai, China). According to manufacturer’s instructions, cells were transfected with the lentiviral particles for 12 h and then screened by puromycin. Western blot analysis was performed to confirm the expression level of P2RX7.

To establish c-Myc knockdown cell lines, cells were incubated with lentiviral vector (hU6-MCS-CMV-BSD) carrying MYC-targeted shRNA (5′-CCUGAGACAGAUCAGCAACAATT-3′), which was purchased from Genechem (Shanghai, China). After incubation for about 12 h, blasticidin was applied to select out positive cells with resistance. Cell lysates were extracted to determine c-Myc expression using western blot.

As for cell lines stably expressing luciferase, the vector Lenti-luciferase-P2A-Neo (Plasmid #105621, Addgene) was used to infect cells, followed by geneticin screening. Cellular luciferase expression level was detected by luciferase assay system (Promega, USA). Even when infected by the same titer of virus, the intensity of luciferase expression may not be consistent in different cell lines. Thus, each cell line was infected by 3 different concentrations of virus and then luciferase expression level was measured and normalized to cell number. Specific proportions of cells from these 3 subgroups were mixed in order to obtain equal normalized luciferase intensity as the corresponding comparison cell line. After such mixing, luciferase assay was performed again and the same number of cells from control and corresponding experimental groups would exhibit essentially identical expression level of luciferase. In this case, mice injected with same number of cells become comparable for bioluminescence imaging in vivo, which can increase the reliability of our results.

### Cell proliferation assay

Cell proliferation was evaluated by Cell Counting Kit-8 assay (CCK-8; Boster, China) according to the manufacturer’s protocol. Briefly, 2.5 × 10^3^ MNNG/HOS and U-2 OS cells were seeded in a 96-well plate and then incubated under certain conditions, including normoxia, hypoxia (1% O2), starvation (2% FBS in culture medium) and chemotherapy administration (cisplatin). More details were shown in specific figure descriptions. At indicated time points, 100 μl basic medium containing 10 μl CCK-8 solution was added to cultured cells in each well. After incubation at 37 °C for 2 h, the optical density (OD) values were recorded by a microplate reader (Bio-Rad, USA) at 450 nm.

### Wound healing migration assay

Cells were seeded and cultured in a 6-well plate until 90% confluence, followed by serum starvation overnight. We used a sterile pipette tip to make a straight scratch, simulating a wound. Subsequently, detached cells were gently washed away and the remaining walled cells continued to be cultured in medium with 2% FBS. At indicated time points, we determined the rate of wound healing by calculating the proportion of wound healing area to the original total wound defect area.

### Matrigel invasion assay

We performed matrigel invasion assay in a 24-well plate with a transwell insert (Corning, USA), which separates the upper and lower compartments by polycarbonate membrane with a pore diameter of 8 μm. The transwell chamber was pre-coated with matrigel (BD Biosciences, USA) in a dilution of 1:8. Then, resuspended cells (2 × 10^4^ cells in 100 μl serum free medium) were well-distributed in the upper compartment. Medium with 10% FBS was added in the lower compartment. After 24 h incubation, cells which successfully traversed chamber membrane were fixed using 4% formalin, followed by 0.1% crystal violet staining. Stained cells were then counted to determine the invasion ability.

### Flow cytometry analysis

Cells (5 × 10^5^) were detached and washed using PBS for cell cycle analysis. Then, cells were fixed and permeabilized by 2 ml of pre-cooled 75% ethanol drop by drop. The cell suspension was vortexed well and maintained at 4 °C overnight. After washing for 2 times, 400 μl PI/RNase Staining Buffer (BD Pharmingen, USA) was added and incubated for 15 min at room temperature in the dark. Samples were detected within 1 h. For cell apoptosis analysis, FITC Annexin V Apoptosis Detection Kit (BD Pharmingen, USA) was used. According to the standard instructions, 1 × 10^5^ cells were resuspended in 100 μl binding buffer and then stained with 5 μl FITC Annexin V and 5 μl PI for 15 min at room temperature in the dark. Another 400 μl binding buffer was added to each tube and analyzed by flowcytometry within 1 h. The single staining of FITC Annexin V or PI was utilized as a control. The detection was carried out by a flow cytometer (BD Biosciences, San Jose, CA, USA) and results were analyzed using FlowJo software (FlowJo, LLC., Ashland, OR, USA).

### Glucose, ATP, pyruvate, LDH and lactate assays

Glucose concentration was determined by O-toluidine method with Glucose Assay Reagent Kit, which was purchased from Beyotime (Shanghai, China). The glucose concentration was measured after cells were cultured for 48 h. The same cultured medium without cell was used as control. We calculated glucose consumption level as the difference between cell cultured and control medium.

Glucose uptake was tested with Glucose Uptake Colorimetric Assay Kit (Biovision, USA) according to the standard protocol. Briefly, 1 × 10^4^ cells were seeded in a 96-well plate and cultured overnight. Cells were then starved for glucose following the pretreatment with Krebs–Ringer-Phosphate-HEPES(KRPH) buffer supplemented with 2% BSA. After incubation with 10 mM 2-DG for 20 min, cells were lysed. The cell lysate was frozen/thawed once, heated up to 85 °C for 40 min, neutralized, and centrifugated at 12,000 rpm for 5 min. The OD value of the supernatant was recorded at 412 nm.

For ATP level assay with ATP Colorimetric Assay Kit (Biovision, USA), a total of 5 × 10^5^ cells were extracted and lysate was then centrifugated. The supernatant was collected for subsequent reaction at room temperature for 30 min. The OD value of reaction mixture was read at 570 nm.

Pyruvate Colorimetric Assay kit (Biovision, USA) was used to estimate pyruvate level. Cells (5 × 10^5^) were extracted to acquire supernatants. After reaction for 30 min, the mixture was measured for OD value at 570 nm. The procedure was similar with ATP assay kit.

With regard to lactate dehydrogenase (LDH) activity assay, 2 × 10^5^ cells were planted in a 6-well plate and collected to obtain supernatants for further reaction. After incubation in the dark for 30 min, the OD value of mixture was detected at 450 nm by a microplate reader.

To estimate lactate production, 1 × 10^5^ cells were seeded in a 12-well plate and incubated overnight. One hour before collection of supernatants, the cultured medium was replaced by serum free medium. Then the reaction mixture was protected from light at room temperature for 30 min. According to Lactate Assay Kit II (Biovision, USA), the lactate production was determined at 450 nm. In addition, lactate within tumor tissue was also measured by using the supernatants from 10 mg tumor tissue homogenate. All above results were normalized to protein concentration.

### Extracellular acidification rate and oxygen consumption rate assays

The extracellular acidification rate (ECAR) and cellular oxygen consumption rate (OCR) were detected by using Seahorse XF Glycolysis Stress Test Kit and Seahorse XF Cell Mito Stress Test Kit (Agilent Technologies, USA) based on their manufacture’s protocols, respectively. Briefly, 2 × 10^5^ cells were seeded in each well and cultured overnight in a Seahorse XF 24 cell culture microplate. Cells could reach up to 95–100% confluence by the next day and thus cell number was almost the same among each group. Both ECAR and OCR were measured with the Seahorse XFe 24 Extracellular Flux Analyzer (Seahorse Bioscience, USA). For ECAR analysis, glucose, oligomycin (oxidative phosphorylation inhibitor) and 2-DG (glycolytic inhibitor) were sequentially added into each well at indicated time points. Similarly, reagents were sequentially injected during OCR test, including oligomycin, p-trifluoromethoxy carbonyl cyanide phenylhydrazone (FCCP, reversible oxidative phosphorylation inhibitor), and rotenone plus antimycin A (Rote/AA, mitochondrial complex I inhibitor and mitochondrial complex III inhibitor). Data were analyzed using Seahorse XF-96 Wave software and normalized to protein weight.

### Transcriptome sequencing (RNA-seq)

The transcriptome sequencing (RNA-seq) was carried out by Novogene (Beijing, China). Total RNA was extracted and purified to obtain mRNA for library generation. The cDNA library was then sequenced on an Illumina Novaseq platform. After quality control of raw data, gene expression level was estimated for each transcript by calculating the value of PFKM, expected number of reads per kilobase of exon model per million base pairs sequenced. A gene was assigned as significantly differentially expressed when its adjusted P value < 0.05 and fold change≥ 2 in the comparison between any two groups. Based on these differentially expressed genes, Gene Ontology (GO) and Kyoto Encyclopedia of Genes and Genomes (KEGG) enrichment analyses were implemented by using the clusterProfiler R package. Additionally, the local version of Gene Set Enrichment Analysis (GSEA) analysis tool (http://www.broadinstitute.org/gsea/index.jsp) was used to perform GSEA analysis for GO data sets.

### Quasi-targeted metabolomics

The quasi-targeted metabolomics was conducted by Novogene (Beijing, China). Briefly, cells were washed with prechilled PBS and incubated with 60% methanol. After centrifugation at 1000 g for 1 min at 4 °C, precipitate was collected and frozen in liquid nitrogen for 15 min. Precooled 80% methanol was added to each sample and the mixture was sonicated for 6 min. High performance liquid chromatography-tandem mass spectrometric (HPLC–MS/MS) analyses were performed using an ExionLC™ AD system (SCIEX). Data generated from HPLC–MS/MS were processed using the SCIEX OS Version 1.4 to integrate and correct the peak. Metabolites were considered to be significantly differential when P value < 0.05 and fold change ≥ 2. The enrichment metabolic pathways with differential metabolites were performed using KEGG database. Moreover, metabolites related to glucose metabolism were analyzed and presented as cluster heatmap.

### Quantitative reverse transcription-PCR (RT-PCR)

Total RNA was extracted using the EZNA Total RNA kit (Omega Bio-Tek, USA) according to manufacturer’s instructions. Then we used 2 ug RNA to synthesize first strand cDNA with Reverse Transcription kit (Toyobo Life Science, Japan). PCR amplification was performed with SYBR Green Real-Time PCR Master Mix (Toyobo Life Science, Japan) on CFX96 (Bio-Rad Laboratories, USA) system. The relative gene expression level was calculated by comparative Ct method and normalized to internal control α-Tubulin. All PCR reactions were repeated in triplicates.

### Western blot analysis

The protein was extracted using radio immunoprecipitation assay lysis buffer (RIPA) containing 1% protease and phosphatase inhibitors (Boster, Wuhan, China). Then we measured the protein concentration by Bicinchoninic Acid Assay (BCA) protein assay kit (Boster, Wuhan, China). A total of 20 μg protein for each sample was separated in a 10% SDS-PAGE gel and subsequently transferred onto a PVDF membrane (Millipore, USA). After blocking with 5% bovine serum albumin (BSA) in Tris-buffered Saline-Tween solution (TBST) for 1 h at room temperature, membranes were incubated with specific primary antibodies after appropriately dilution at 4 °C overnight. The membrane was rinsed in Tris-buffered saline with 0.1% Tween-20 (TBST) for 3 times, followed by incubation with horse-radish peroxidase (HRP)-conjugated secondary antibodies (Sigma-Aldrich, USA) for 1 h at room temperature. After washing, blots were finally visualized under enhanced chemiluminescence (ECL) System (Thermo Fisher Scientific, USA). Protein expression level was semi-quantified by Image Lab system version 5.1 (Bio-Rad Laboratories, USA) and normalized to corresponding α-Tubulin (total and cytoplasm protein) or Lamin B (nuclear protein) expression. The primary antibodies used in this study included anti-P2RX7 (ab48871, Abcam), anti-SLC2A1 (#12939, Cell Signaling Technology), anti-HK2 (#2106, Cell Signaling Technology), anti-PFKP (#8164, Cell Signaling Technology), anti-ALDOA (#3188, Cell Signaling Technology), anti-GAPDH (#2118, Cell Signaling Technology), anti-PGK1 (#68540, Cell Signaling Technology), anti-PGAM1 (#12098, Cell Signaling Technology), anti-ENO1 (#3810, Cell Signaling Technology), anti-PKM (#3190, Cell Signaling Technology), anti-LDHA (#3582, Cell Signaling Technology), anti-PDHA1 (#3205, Cell Signaling Technology), anti-DLAT (#12362, Cell Signaling Technology), anti-DLD (ab133551, Abcam), anti-CS (ab96600, Abcam), anti-ACO1 (#20272, Cell Signaling Technology), anti-IDH2 (#56439, Cell Signaling Technology), anti-OGDH (ab137773, Abcam), anti-SUCLG1 (ab97867, Abcam), anti-SDHA (#11998, Cell Signaling Technology), anti-FH (#4567, Cell Signaling Technology), anti-CDK4 (#12790, Cell Signaling Technology), anti-PCNA (#2586, Cell Signaling Technology), anti-MMP2 (#87809, Cell Signaling Technology), anti-MMP9 (#13667, Cell Signaling Technology), anti-c-Myc (ab32072, Abcam), c-MycS62 (ab185656, Abcam), c-MycT58 (ab185655, Abcam), anti-Tubulin (ab52866, Abcam), and anti-Lamin B1 (ab16048, Abcam). All experiments were independently repeated for three times and the representative images were presented.

### Immunofluorescence

Cells were washed with PBS for 3 times, fixed with 4% paraformaldehyde for 15 min and permeabilized with 0.5% Triton X-100 for another 15 min. Then, goat serum was added to block non-specific antigens at room temperature for 30 min. Cells were incubated overnight with suitably diluted primary antibodies at 4 °C. Fluorescence labeled secondary antibody solutions were subsequently added and incubated for 1 h in dark. In addition, nuclei were counterstained by DAPI. A fluorescent microscopy (EVOS FL Auto Imaging System, Life technologies, Gaithersburg, MD) was used for observation and image capture.

### Mouse xenograft model

Female BALB/c nude mice (4 weeks old) were purchased from the Experimental Animal Center of Huazhong University of Science and Technology (Wuhan, China). All animal experiments were performed in accordance with institutional guidelines for the care of laboratory animals and approved by the Ethics Committee of Huazhong Science and Technology University. For orthotopic tumor model, 2 × 10^6^ MNNG/HOS-luciferase cells suspended in 50 μl PBS were injected into the intramedullary cavity in left tibia by a 25-gauge needle. Four weeks after injection, luciferase expression of tumor xenograft was detected by an IVIS Lumina Imaging System. In addition, three mice from each group with average-sized tumors were selected for PET imaging. Subsequently, all mice were sacrificed and tumors were harvested. We measured the weight of tumor tissue and also counted the number of metastatic lung nodules. Then, tumor tissues were fixed in 4% paraformaldehyde for further histological analyses.

To establish a subcutaneous tumor model, 100 μl PBS suspension containing 5 × 10^6^ MNNG/HOS cells was subcutaneously injected into the right armpit region. After the tumor was macroscopically visible (around 1 week), mice burdened with P2RX7 overexpression or control cells were randomly divided into two groups (n = 6 per group), and treated with PBS or 2-DG (500 mg/kg) intraperitoneally every other day. Tumor growth was recorded every 3 days after 1 week by vernier caliper and tumor volume was calculated based on the following formula: volume = π/6 × L x W^2^, where L represents the longest diameter of tumor and W the shortest. Mice were sacrificed 4 weeks after cell injection. Tumors were dissected and weighed.

### Bioluminescence imaging

Mice were intraperitoneally injected with D-Luciferin (115144-35-9, GoldBio, USA) and then anesthetized using pentobarbital sodium (50 mg/kg). Ten minutes after injection, bioluminescence images were acquired by IVIS 200 Xenogen system (IVIS Spectrum; PerkinElmer, Waltham, USA) and analyzed using Living Image 4.1 software (Xenogen, Caliper Life Sciences).

### ^18^F-FDG PET/CT

Mice were fasted for 8 h prior to PET/CT examination. Each mouse was intraperitoneally injected with 1.85 MBq ^18^F-FDG. Forty minutes after injection, mice were anesthetized and fixed on the testing table. At the time point, one hour post injection, we collected static images over a fixed 10-min acquisition by using animal PET/CT miniEXPLORER (united imaging, China). Along with PET scan, CT images were also obtained for anatomic reference. To generate more high-resolution images, all data were reconstructed through a three-dimensional ordered subsets expectation maximization (OSEM) algorithm (4 OSEM iterations, requested resolution: 0.6 mm). Besides, scatter, attenuation, and decay corrections were also applied. Then, we recorded maximum standard uptake value (SUVmax) and mean SUV (SUVmean) to evaluate tracer uptake within tumors.

### Histological analysis

Tumor specimens were fixed in 4% paraformaldehyde solution for 2 days and then embedded in paraffin blocks. Slices with 5 μm thickness were prepared for subsequent experiments, including hematoxylin and eosin (HE) and immunohistochemistry (IHC) analyses. Briefly, the paraffin sections were gradient dewaxed and washed in water. With regard to HE staining, the slices were incubated in hematoxylin for 5 min, followed by eosin solution for another 5 min. After dehydration, slices were sealed by neutral gum for storage and observation. We also performed IHC staining with Ki67 antibody. Tissue section was heated in citric acid antigen repair buffer (pH 6.0) by using a microwave oven for 20 min. After antigen retrieval, section was immersed in 3% (w/v) hydrogen peroxide solution (H_2_O_2_) for 25 min and blocked with 5% BSA solution for 30 min. Subsequently, section was incubated with anti-Ki67 antibody (ab16667, Abcam) in a humidified box at 4 °C overnight. Then horseradish peroxidase (HRP)-conjugated secondary antibody was added to cover each slice. After incubation for 1 h at room temperature, color reaction was developed using freshly prepared 3, 3-diaminobenzidiine tetrahydrochloride (DAB) solution and terminated by water under microscopic monitoring. After nuclei counterstaining by hematoxylin for 3 min, slice was sealed as above HE staining.

### Statistical analysis

All data were presented as mean ± standard deviation (SD) and analyzed by GraphPad Prism (Version 9.3.1). Student’s t-test was used to compare the statistical differences between two groups, while one-way analysis of variance (ANOVA) followed by Tukey HSD test was performed for comparisons among multiple groups. If time was involved as an independent factor, then we selected two-way ANOVA test. P value less than 0.05 was considered statistically significant.

## Results

### Identification of P2RX7 as a key regulator of metabolic reprogramming in osteosarcoma

**Fig. 1:**
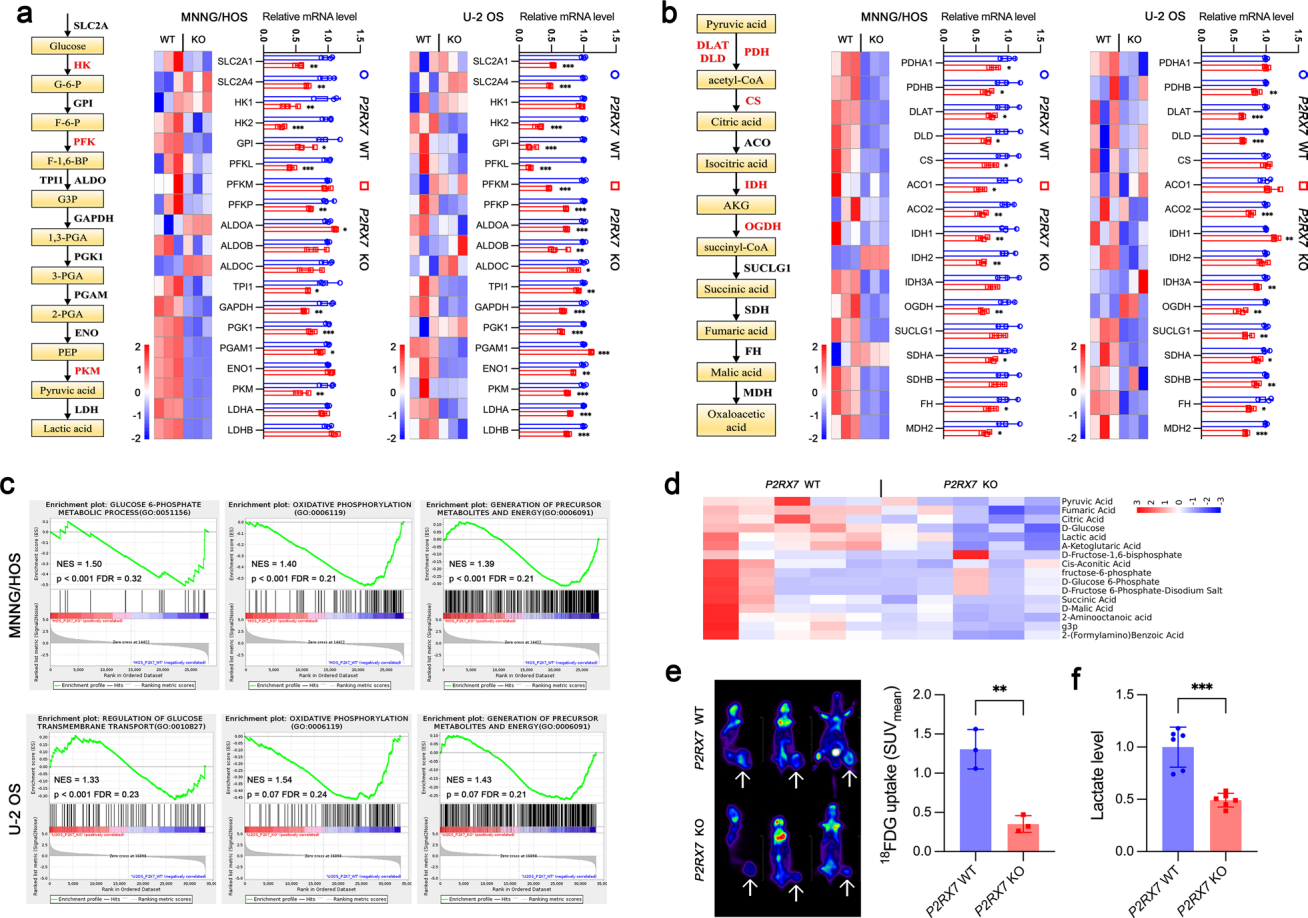
P2RX7 regulated glucose metabolism in osteosarcoma.** a** Heatmap of glycolysis-related gene expression generated by RNA-seq in *P2RX7* wild-type (WT) and knockout (KO) cells, respectively. qRT-PCR further confirmed the differential expression of glycolytic genes between *P2RX7* WT and KO cells. **b** Heatmap of oxidative phosphorylation-related gene expression analyzed by RNA-seq in *P2RX7* WT and KO cells, respectively. qRT-PCR further confirmed the differential expression of these genes between *P2RX7* WT and KO cells. **c** Gene Set Enrichment Analysis (GSEA) analysis suggested significant difference of metabolic pathways between *P2RX7* WT and KO cells. **d** Clustering heatmap of glucose metabolites by Quasi-targeted metabolomics between *P2RX7* WT and KO MNNG/HOS cells. **e** Glucose uptake in tibia osteosarcoma through ^18^F-FDG PET/CT detection. Arrows indicated tumor glucose uptake. **f** Lactate level of tumor tissues from mice injected with *P2RX7* WT and KO MNNG/HOS cells. Data are shown as mean ± standard deviation (SD). *p < 0.05, **p < 0.01, ***p < 0.001 versus corresponding *P2RX7* WT group

Our previous study [40] has suggested an essential role of P2RX7 in OS progression. However, the underlying mechanisms remain to be further explored. In this study, we performed RNA sequencing (RNA-seq) on *P2RX7* knockout (KO) OS cells and wild type (WT) control cells, including MNNG/HOS and U-2 OS cell lines. We identified a total of 2290 and 1010 significantly differentially expressed genes in MNNG/HOS and U-2 OS cells, respectively. Clustering analysis revealed a shift in the expression pattern of genes related to glucose metabolism in *P2RX7* KO cells. In terms of glycolysis, clustering heatmap indicated that glycolysis-related genes were differentially expressed. Compared to WT cells, qRT-PCR analysis further confirmed that *P2RX7* KO dramatically reduced gene expressions of solute carrier family 2 member 1 (SLC2A1), SLC2A4, hexokinase 1 (HK1), HK2, glucose-6-phosphate isomerase (GPI), phosphofructokinase, liver type (PFKL), PFK platelet (PFKP), triosephosphate isomerase 1 (TPI1), glyceraldehyde-3-phosphate dehydrogenase (GAPDH), phosphoglycerate kinase 1 (PGK1), phosphoglycerate mutase 1 (PGAM1), enolase 1 (ENO1) and pyruvate kinase M (PKM), but not PFK muscle (PFKM), aldolase A (ALDOA), ALDOB, ALDOC, lactate dehydrogenase A (LDHA) and LDHB in MNNG/HOS cells, and all of these genes except HK1 and PGAM1 in U-2 OS cells (Fig. 1a). The primers of these genes used in this study were listed in Additional file 1: Table S1. With regard to oxidative phosphorylation, similar results were observed in cluster heatmap. The altered expressions of associated genes were further validated by qRT-PCR at mRNA level. *P2RX7* KO cells showed a significant reduction in gene expressions, including pyruvate dehydrogenase E1 subunit alpha 1 (PDHA1), PDHB, dihydrolipoamide S-acetyltransferase (DLAT), dihydrolipoamide dehydrogenase (DLD), citrate synthase (CS), aconitase 1 (ACO1), ACO2, isocitrate dehydrogenase (NADP( +)) 1 (IDH1), IDH2, oxoglutarate dehydrogenase (OGDH), succinate dehydrogenase complex flavoprotein subunit A (SDHA), SDHB, fumarate hydratase (FH) and malate dehydrogenase 2 (MDH2), but not IDH3A and succinate-CoA ligase GDP/ADP-forming subunit alpha (SUCLG1) in MNNG/HOS cells. Additionally, all of these oxidative phosphorylation related genes were significantly downregulated except PDHA1, CS, ACO1, IDH1 and IDH2 in U-2 OS *P2RX7* KO cells (Fig. 1b). Consistently, Gene Set Enrichment Analysis (GSEA) implied that glucose metabolic capacity appeared to be reduced in both MNNG/HOS and U-2 OS cells (Fig. 1c). Moreover, metabolomics was carried out focusing on metabolites related to glucose metabolism and energy production, which were negatively modulated in *P2RX7* KO cells as shown in Fig. 1d. Kyoto Encyclopedia of Genes and Genomes (KEGG) analysis revealed that oxidative phosphorylation was included in the top 20 enrichment pathways (Additional file 2: Figure S1a). In a mouse model of osteosarcoma in situ, P2RX7 obviously improved glucose uptake (Fig. 1e) and lactate production (Fig. 1f) within tumor tissue. These data demonstrated the critical role of P2RX7 in regulation of glucose metabolism in OS.

### P2RX7 elevated glucose metabolism capacity and related protein expression in osteosarcoma cells

**Fig. 2:**
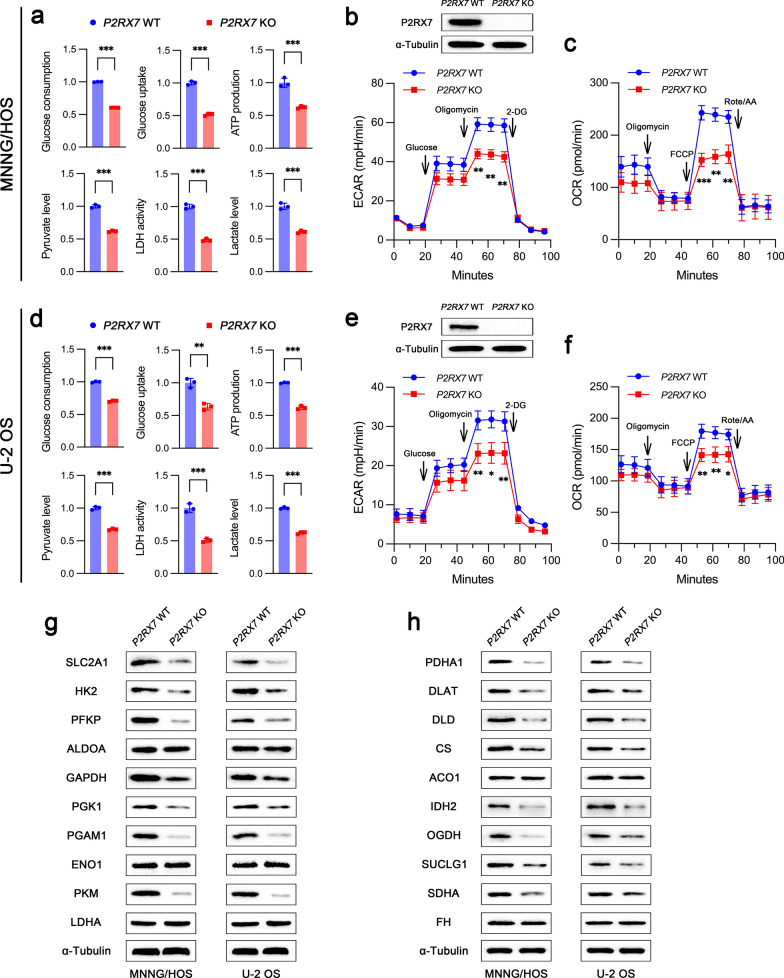
P2RX7 enhanced glycolysis and oxidative phosphorylation.** a**, **d** Glucose consumption, glucose uptake, ATP production, pyruvate level, LDH activity and lactate level were determined in *P2RX7* wild-type (WT) and knockout (KO) MNNG/HOS cells (**a**), as well as in U-2 OS cells (**d**). **b**, **e** The analysis of extracellular acidification rate (ECAR) in MNNG/HOS cells (**b**) and U-2 OS cells (**e**), respectively. **c**, **f** The analysis of oxygen consumption rate (OCR) in MNNG/HOS cells (**c**) and U-2 OS cells (**f**), respectively. **g** Representative immunoblots of glycolytic proteins in *P2RX7* WT and KO osteosarcoma cells. **h** Representative immunoblots of oxidative phosphorylation-related proteins in *P2RX7* WT and KO osteosarcoma cells. Data are shown as mean ± standard deviation (SD). *p < 0.05, **p < 0.01, ***p < 0.001 versus corresponding *P2RX7* WT group

Since P2RX7 could regulate gene expression related to glucose metabolism, we thus tested whether there was a reconstruction of biological processes associated with glucose metabolism. Generally, glucose is absorbed into cytoplasm and catabolized into pyruvate, which will be further processed into lactate or transported into the mitochondria for oxidative phosphorylation, accompanied by ATP production to supply energy. In both MNNG/HOS (Fig. 2a) and U-2 OS cells (Fig. 2d), P2RX7 obviously facilitated glucose consumption and uptake, increased ATP production, and elevated levels of metabolites such as pyruvate and lactate. LDH activity was also higher in WT cells compared with *P2RX7* KO cells. Besides, we determined a remarkable attenuation of extracellular acidification rate (ECAR), an indicator of glycolysis flux, in *P2RX7* KO cells (Fig. 2b). Oxygen consumption rate (OCR), reflecting mitochondrial oxidative respiration, was also determined to decrease caused by *P2RX7* knockout (Fig. 2c). A significant downregulation of ECAR (Fig. 2e) and OCR (Fig. 2f) by *P2RX7* knockout was also observed in U-2 OS cells. Immunoblot analysis demonstrated an overall decrease in the expression of proteins, which are enzymes involved in glycolytic reactions, in both MNNG/HOS and U-2 OS *P2RX7* KO cells (Fig. 2g). In particular, key enzymes mediating irreversible reactions, such as HK2, PFKP and PKM, were all significantly reduced. Similar immunoblot results were also obtained regarding oxidative phosphorylation, which showed that key enzymes including PDHA1, DLAT, DLD, CS, IDH2 and OGDH were markedly downregulated by *P2RX7* knockout (Fig. 2h).

### P2RX7 enhanced cell proliferation, migration, invasion and chemo-resistance in vitro

**Fig. 3:**
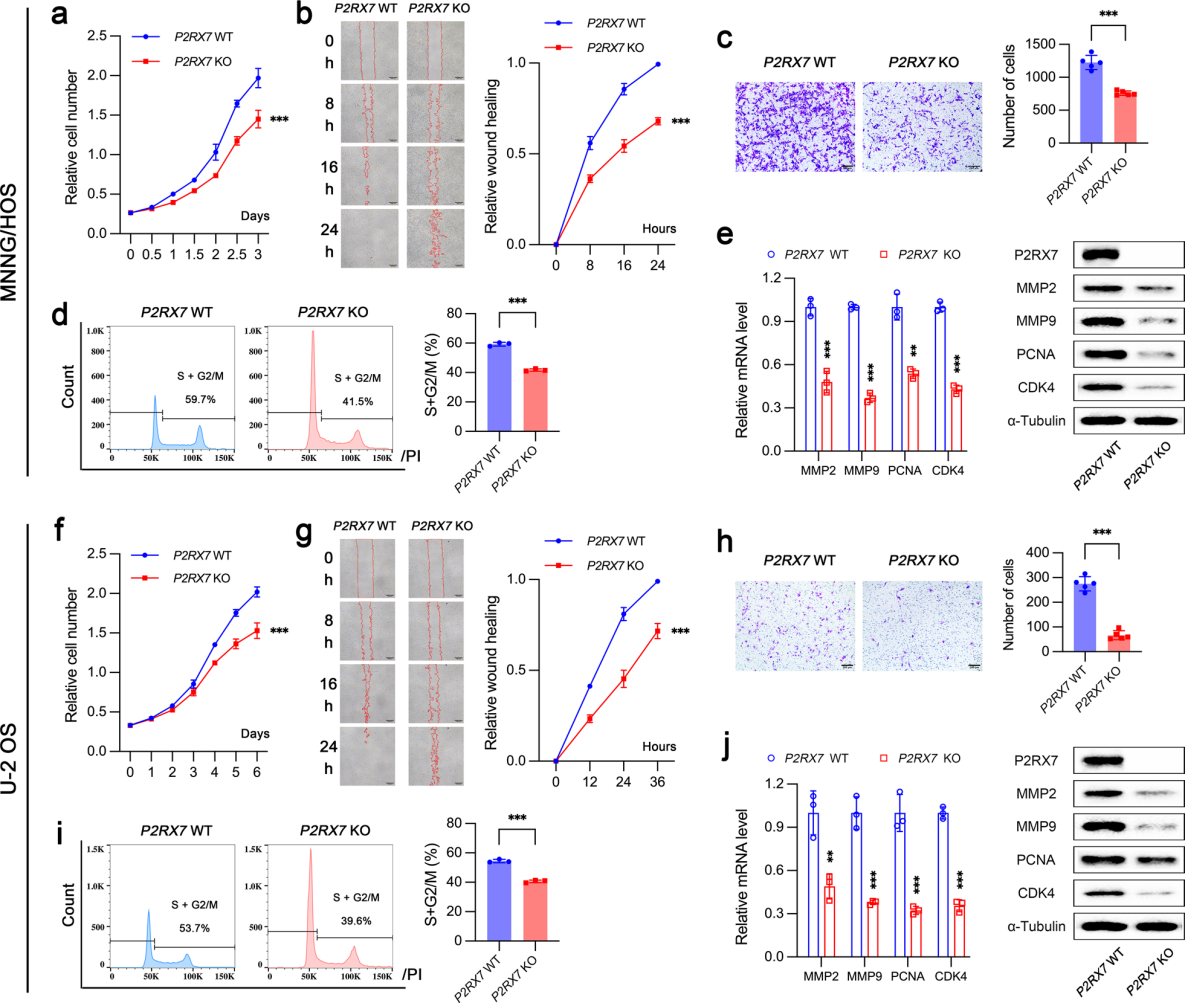
P2RX7 promoted proliferation, migration and invasion of osteosarcoma cells in vitro. **a**, **f** Proliferation of osteosarcoma cells analyzed by CCK8. **b**, **g** Wound healing assay was performed to evaluate cell migration in *P2RX7* wild -type (WT) and knockout (KO) cells. **c**, **h** The invasiveness of osteosarcoma cells with *P2RX7* knockout or not. **d**, **i** Cell cycle analysis by flowcytometry in *P2RX7* WT and KO cells. **e**, **j** The expressions of proliferation-related and invasion-related genes assessed by qRT-PCR and immunoblot analyses. Data are shown as mean ± standard deviation (SD). *p < 0.05, **p < 0.01, ***p < 0.001 versus corresponding *P2RX7* WT group

The effects of P2RX7 on cell biological behaviors associated with tumorigenesis and progression were evaluated in OS cells. *P2RX7* knockout obviously impaired cell proliferation in both MNNG/HOS (Fig. 3a) and U-2 OS cells (Fig. 3f). Wound healing assay indicated that P2RX7 could prominently facilitate OS cell migration (Fig. 3b and g). The invasiveness ability of OS cell was also largely suppressed when P2RX7 was knocked out (Fig. 3c and h). In order to explore the potential mechanisms underlying cell proliferation regulated by P2RX7, we analyzed cell cycle using flow cytometry. The results demonstrated that P2RX7 accelerated cell cycle transition to cell division (Fig. 3d and i) and thus enhanced cell proliferation. Consistently, cell cycle and DNA replication were covered in the top 20 enrichment pathways by both Gene Ontology (GO) (Additional file 2: Fig. S1b) and KEGG analyses (Additional file 2: Fig. S1c). Moreover, GO pathways related to extracellular matrix and collagen catabolic process were ranked among the top 20 enrichment pathways (Additional file 2: Fig. S1b). In addition, pathways involving cell cycle and DNA replication were negatively regulated in *P2RX7* KO groups through GSEA data analysis (Additional file 2: Fig. S1d, e). We also found that *P2RX7* knockout significantly decreased the expression of proliferative genes (proliferating cell nuclear antigen (PCNA) and cyclin dependent kinase 4 (CDK4)) and metastatic genes (matrix metallopeptidase 2 (MMP2) and MMP9) at mRNA and protein levels (Fig. 3e and j). Chemoresistance, a property commonly found in tumor cells, is one of the most important factors contributing to tumor treatment failure. In our study, we suggested that P2RX7 depletion could substantially reduce the half maximal inhibitory concentration (IC50) of cisplatin (CDDP) in MNNG/HOS cells from 4.23 μM to 2.57 μM (Additional file 3: Fig. S2a). Flowcytometry analysis was further performed to investigate cell survival under CDDP stress. P2RX7 knockout significantly increased the sensitivity of MNNG/HOS cells to CDDP and apoptosis (Additional file 3: Fig. S2b). Taken together, these data demonstrated that P2RX7 is critical for tumorigenesis and progression of OS.

### P2RX7 promoted cell proliferation, migration, invasion and glucose metabolism under adverse conditions

Considering that hypoxia is a key feature of cancer, including OS, we examined whether P2RX7 plays a role in the regulation of cell biological behaviors and glucose metabolism under hypoxia. Consistent with the results under normoxia, *P2RX7* knockout prominently inhibited cell proliferation (Additional file 4: Fig. S3a and f), migration (Additional file 4: Fig. S3b and S3g) and invasion (Additional file 4: Fig. S3c and h). In addition, the glucose metabolism capacity of *P2RX7* KO cells was also repressed, such as reduction of glucose consumption and uptake, lactate and ATP production (Additional file 4: Fig. S3d and i). Since rapid tumor growth requires adequate energy, energy supply becomes relatively insufficient in tumor tissues, especially in tumor central region. Thus, we further demonstrated the promoting effect of P2RX7 on cell proliferation in the case of serum starvation (Additional file 4: Fig. S3e and j).

### P2RX7 promoted tumor growth and lung metastasis in vivo

**Fig. 4:**
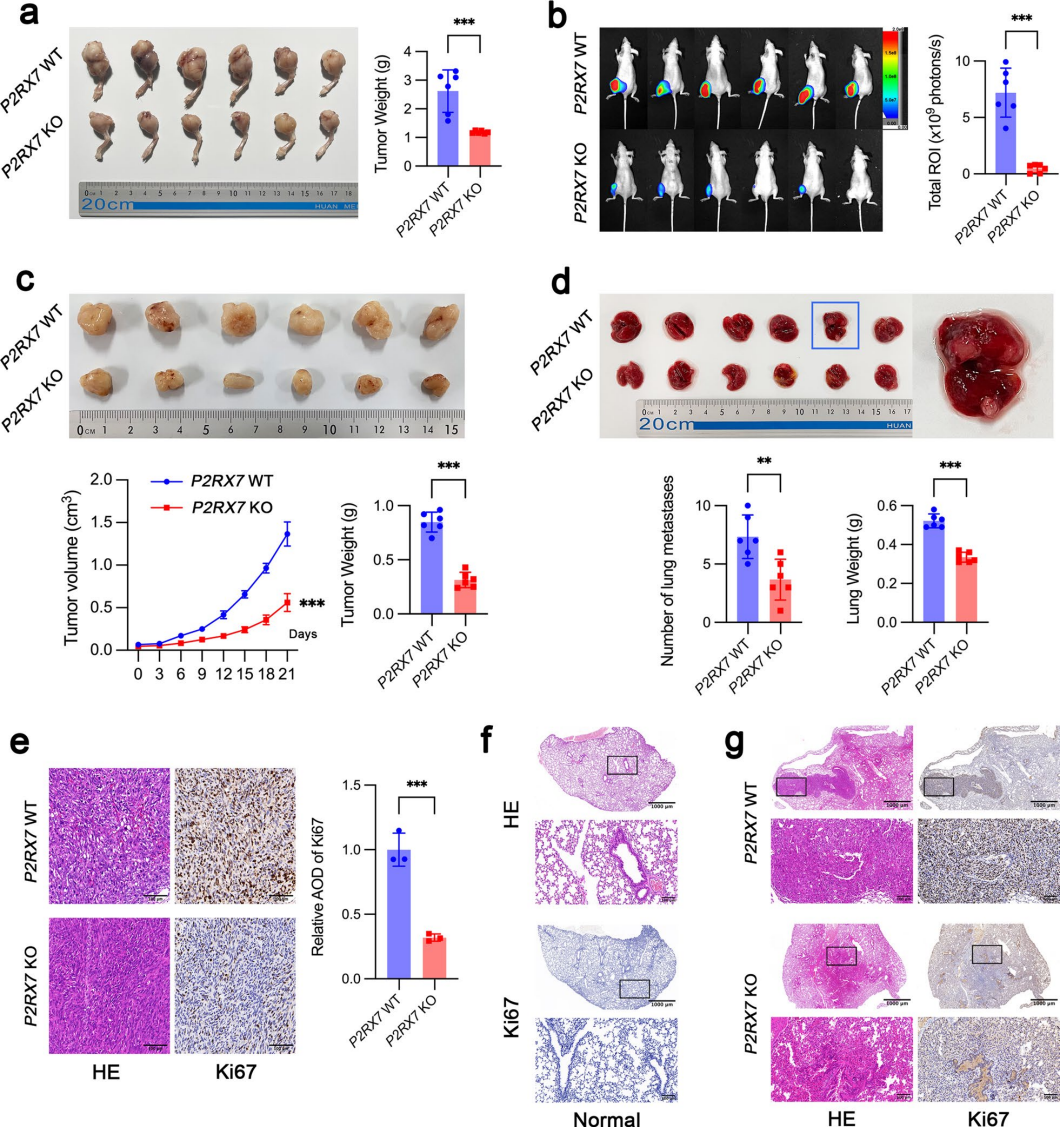
P2RX7 facilitated osteosarcoma growth and lung metastasis through accelerating glucose metabolism in vivo.** a** *P2RX7* wild-type (WT) and knockout (KO) MNNG/HOS cells were injected into mouse tibia to establish orthotopic osteosarcoma model. Tumor weights were recorded after mice sacrifice. **b** Bioluminescence images of tumor-bearing mice. The total luminous intensity of the region of interest (ROI) was presented. **c** Subcutaneous tumor was established and growth curve was plotted. Tumor were weighed after isolation. **d** Lung tissue was resected and metastatic nodules were counted. Lung weights were also measured. **e** Representative images of histological analysis, including Hematoxylin and Eosin (HE) staining and immunohistochemistry (IHC) with Ki67 antibody. Average optical density (AOD) of IHC was calculated and compared. **f** HE and IHC stainings in normal lung tissue.** g** Histological analysis for lung tissue with metastasis by HE and IHC. Data are shown as mean ± standard deviation (SD). *p < 0.05, **p < 0.01, ***p < 0.001 versus corresponding *P2RX7* WT group

In order to test the role of P2RX7 in tumor growth and lung metastasis, we established an orthotopic OS model with spontaneous metastasis in nude mice by injecting *P2RX7* WT and KO MNNG/HOS cells into tibia. Tumor with WT cells grew significantly faster compared to tumor with corresponding KO cells (Fig. 4a). In vivo bioluminescence imaging further validated this effect of P2RX7 (Fig. 4b). Additionally, similar results were observed in a subcutaneous tumor model (Fig. 4c). It is well known that lung is the most common site of OS metastasis. Therefore, spontaneous lung metastasis was evaluated in nude mice with orthotopic OS. Mice injected with *P2RX7* KO cells showed a substantial reduction in the number of lung metastases and the weight of lung tissue (Fig. 4d). By IHC staining, it was demonstrated that *P2RX7* KO could dramatically inhibit Ki67 expression in vivo (Fig. 4e), an indicator of tumor growth. As shown in Fig. 4f, normal lung tissue had almost no Ki67-positive regions. In contrast, lung tissue with metastatic nodules presented intense Ki67 staining (Fig. 4g).

### P2RX7 accelerated cell proliferation and tumor growth partially through metabolic reprogramming

As expected, both glycolysis inhibitor (2-DG) and oxidative phosphorylation inhibitor (oligomycin) could significantly inhibit cell proliferation (Additional file 5: Fig. S4a). Importantly, 2-DG and oligomycin reduced the ability of P2RX7 to promote cell proliferation (Additional file 5: Fig. S4b). We further tested whether P2RX7 regulated tumor growth via glucose metabolism in nude mice. As shown in Additional file 5: Fig. S4c, P2RX7 overexpression facilitated tumor growth, which could be to some extent abolished by 2-DG. These data suggested that P2RX7 promoted cell proliferation and tumor growth partially through metabolic remodeling.

### P2RX7 enhanced c-Myc expression and stabilization

**Fig. 5:**
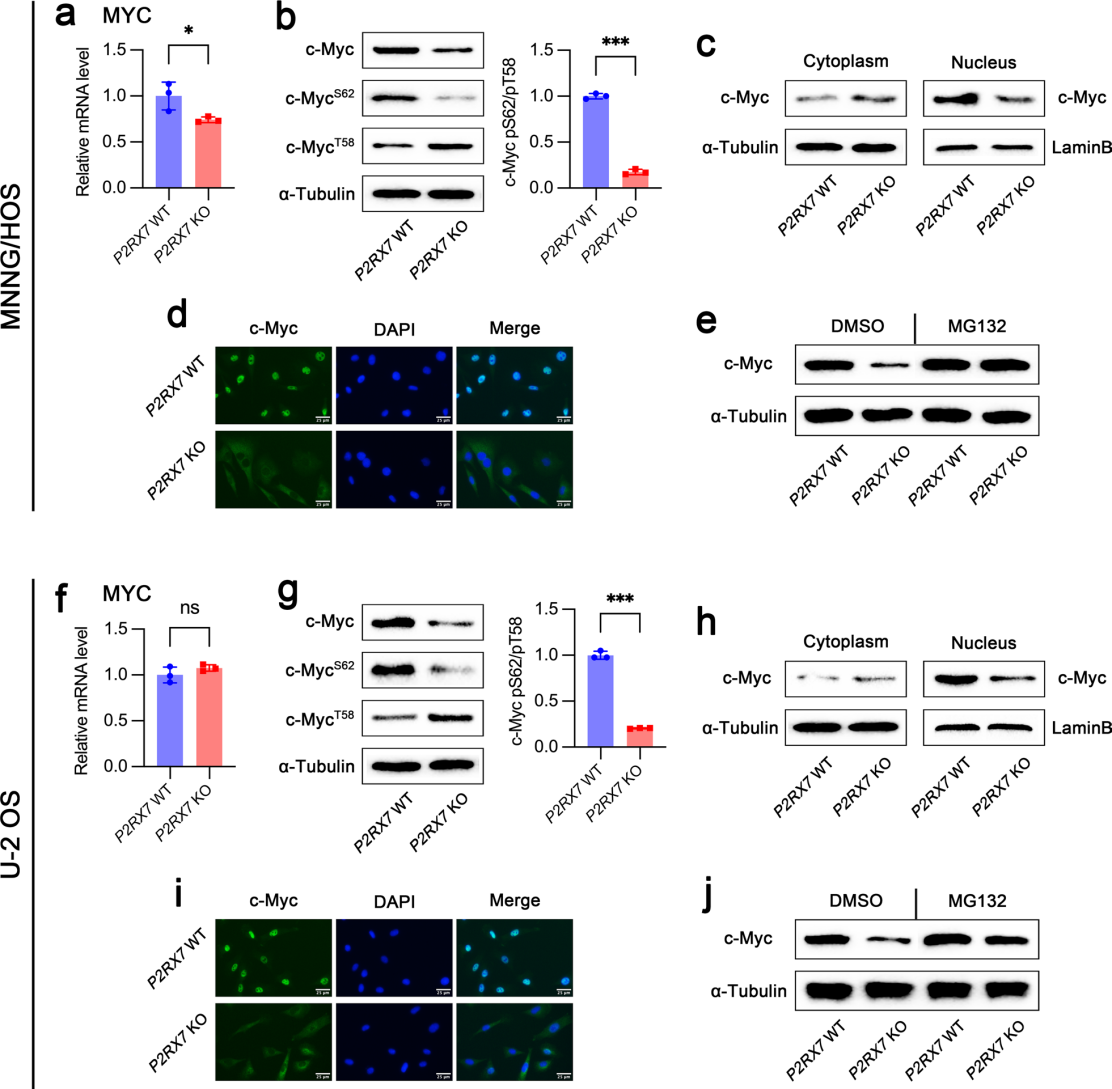
P2RX7 promoted the stabilization of c-Myc via increasing nuclear retention and decreasing ubiquitination-mediated degradation.** a**, **f** The effect of P2RX7 on *MYC* gene expression assessed by qRT-PCR in osteosarcoma cells. **b**, **g** Immunoblot analysis was performed to estimate total c-Myc, phosphorylated c-Myc^S62^ and c-Myc^T58^ protein levels and the ratio of pS62/pT58 was then calculated. **c**, **h** Immunoblot of c-Myc expression in cytoplasm and nucleus. Lamin B and α-tubulin were used as internal controls for nuclear and cytoplasmic lysates, respectively. **d**, **i** Immunofluorescence showed the expression pattern of c-Myc in *P2RX7* wild-type (WT) and knockout (KO) cells. Nuclei were counterstained with DAPI. **e**, **j** c-Myc expression was evaluated by immunoblot in *P2RX7* WT and KO cells treated with DMSO or ubiquitination inhibitor MG132 (10 μM, 2 h). Data are shown as mean ± standard deviation (SD). *p < 0.05, **p < 0.01, ***p < 0.001 versus WT counterpart

Recently, MYC has gained increasing attention as a key regulator involved in cancer metabolism. Due to the prominent effect of P2RX7 on glucose metabolism in OS, we therefore hypothesized a possible association between P2RX7 and c-Myc. Markedly reduced expression of c-Myc was found in MNNG/HOS *P2RX7* KO cells at both mRNA (Fig. 5a) and protein levels (Fig. 5b). Although there was no significant difference in c-Myc expression at mRNA level (Fig. 5f), c-Myc protein was clearly decreased in U-2 OS *P2RX7* KO cells (Fig. 5g). In general, phosphorylation of serine-62 (pS62) induces c-Myc to enter the nucleus for its transcriptional regulatory function, whereas phosphorylation at threonine-58 (pT58) leads to its transportation into cytoplasm, followed by ubiquitination-dependent protein degradation [49]. As expected, pS62 c-Myc was dramatically decreased and pT58 c-Myc increased when *P2RX7* was knocked out in both MNNG/HOS (Fig. 5b) and U-2 OS cells (Fig. 5g). In the above two cell lines, the ratio of pS62/pT58 c-Myc dropped to around 0.18 and 0.20 fold, respectively, suggesting reduced stabilization of c-Myc. Importantly, the accumulation of c-Myc in nucleus was largely hindered by *P2RX7* knockout (Fig. 5c and h). Consistent with the results of immunoblotting, immunofluorescence also displayed an altered pattern of c-Myc expression, particularly strongly enhanced expression within nuclei (Fig. 5d and i). We further explored whether ubiquitination-dependent proteolysis in proteasome participated in P2RX7-mediated c-Myc stabilization. Importantly, administration of the proteasome inhibitor MG132 remarkably abolished *P2RX7* knockout-induced c-Myc degradation (Fig. 5e and j). Besides, data from GESA analysis inferred that regulatory pathways related to protein ubiquitination were also enriched with statistical significance when comparing *P2RX7* WT and KO cells (Additional file 2: Fig. S1f). These findings revealed that P2RX7 enhanced c-Myc stability through promoting nuclear retention and impairing ubiquitination-dependent degradation.

### P2RX7 regulated glucose metabolism and cell biological behaviors through c-Myc

**Fig. 6:**
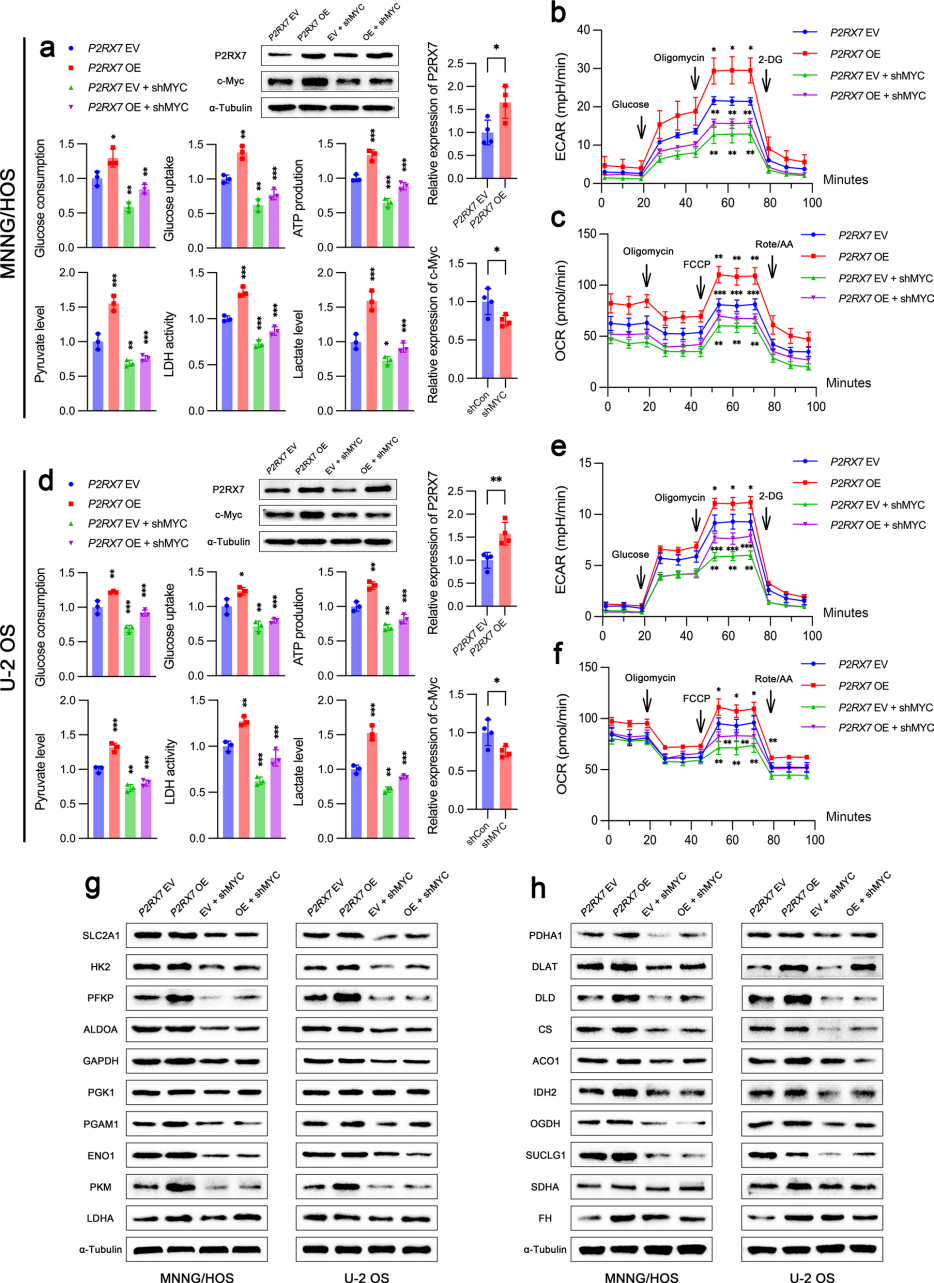
P2RX7 enhanced glucose metabolism via c-Myc.** a**, **d** Glucose consumption, glucose uptake, ATP production, pyruvate level, LDH activity and lactate level were examined in MNNG/HOS cells (**a**) and U-2 OS cells (**d**) transfected with empty vector (*P2RX7* EV), *P2RX7* plasmid (P2RX7 *OE*), EV plus *MYC* shRNA (*P2RX7* EV + shMYC) and OE plus shMYC (*P2RX7* OE + shMYC). **b, e** MNNG/HOS and U-2 OS cells were transfected as in **a** and extracellular acidification rate (ECAR) was then analyzed. **c**, **f** MNNG/HOS and U-2 OS cells were transfected as in **a** and oxygen consumption rate (OCR) was then determined. **g** Representative immunoblots of glycolytic protein expressions in osteosarcoma cells transfected as in **a**. **h** Representative immunoblots of oxidative phosphorylation-related protein expressions in osteosarcoma cells transfected as in **a**. Data are shown as mean ± standard deviation (SD). *p < 0.05, **p < 0.01, ***p < 0.001 versus corresponding *P2RX7* EV or OE group

**Fig. 7:**
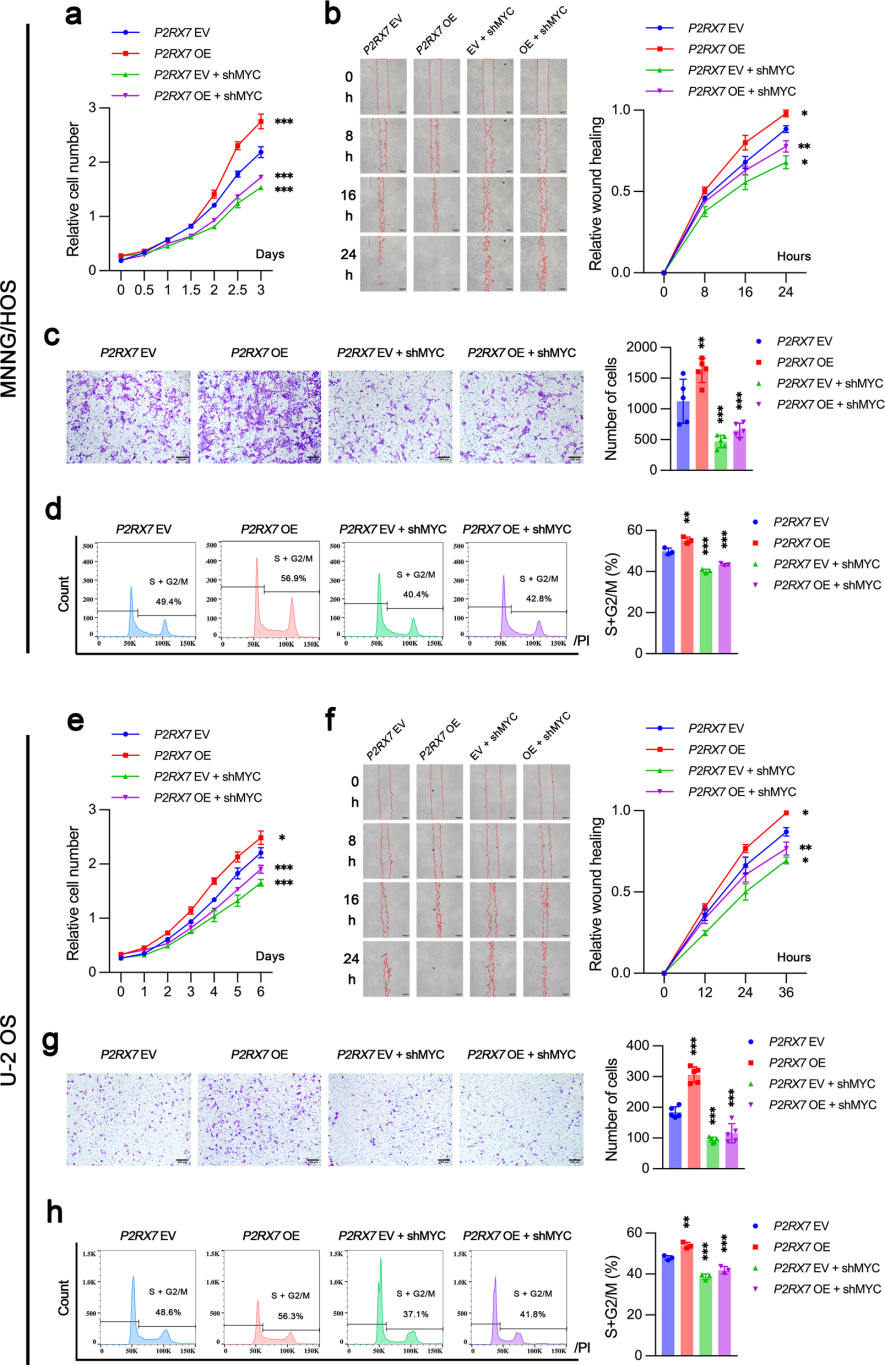
P2RX7 promoted proliferation, migration and invasion of osteosarcoma cells via c-Myc in vitro. **a**, **e** Osteosarcoma cells were transfected with empty vector (*P2RX7* EV), *P2RX7* plasmid (*P2RX7* OE), EV plus *MYC* shRNA (*P2RX7* EV + shMYC) and OE plus shMYC (*P2RX7* OE + shMYC), respectively. P2RX7-mediated promotion of cell proliferation was attenuated by c-Myc knockdown in osteosarcoma cells. **b**, **f** P2RX7-mediated acceleration of cell migration was suppressed by c-Myc knockdown in osteosarcoma cells. **c**, **g** c-Myc knockdown impaired P2RX7-induced invasiveness of osteosarcoma cells. **d**, **h** c-Myc knockdown reduced the ability of P2RX7 to promote cell cycle transition of osteosarcoma cells. Data are shown as mean ± standard deviation (SD). *p < 0.05, **p < 0.01, ***p < 0.001 versus corresponding *P2RX7* EV or OE group

In order to examine the hypothesis that c-Myc was responsive to the function of P2RX7 in the regulation of glucose metabolism, we constructed cell lines overexpressing P2RX7 with or without transfection of *MYC* shRNA. We first investigated the efficiency of P2RX7 overexpression and c-Myc knockdown by western blot analysis. P2RX7 overexpression significantly increased glucose consumption and uptake, ATP production, pyruvate and lactate levels and LDH activity, all of which could be largely blocked by c-Myc knockdown (Fig. 6a and d). Furthermore, c-Myc downregulation virtually abrogated the promoting effects of P2RX7 on both ECAR (Fig. 6b and e) and OCR (Fig. 6c and f), which reflect glycolysis and oxidative phosphorylation capacity, respectively. Besides, P2RX7 upregulated the expression of the majority of glycolysis-related genes in a predominantly c-Myc dependent manner (Additional file 6: Fig. S5a and c). Similar changes were also observed in the expression of oxidative phosphorylation related-genes (Additional file 6: Fig. S5b and d). At protein level, immunoblot analysis showed a trend of decreased glucose metabolism-related proteins in c-Myc knockdown cells (Fig. 6g and h). Notably, the remarkable upregulation of HK2, PFKP, PGAM1, PKM, DLAT, DLD, IDH2 and OGDH proteins, stimulated by P2RX7, was greatly reduced by c-Myc knockdown. These results indicated that c-Myc played a pivotal role in P2RX7-mediated metabolic reprogramming. We also found that c-Myc knockdown largely attenuated the ability of P2RX7 to promote cell proliferation (Fig. 7a and e), migration (Fig. 7b and f) and invasion (Fig. 7c and g). Additionally, P2RX7 overexpression dramatically accelerated cell cycle transition, which was greatly impeded by c-Myc downregulation (Fig. 7d and h). Moreover, P2RX7 overexpression largely suppressed CDDP-induced cell apoptosis, while c-Myc knockdown almost abrogated this effect (Additional file 3: Fig. S2c). These results demonstrated that P2RX7 regulated glucose metabolism and cell biological behaviors predominantly through c-Myc.

### P2RX7 promoted tumor growth and lung metastasis via c-Myc in vivo

**Fig. 8:**
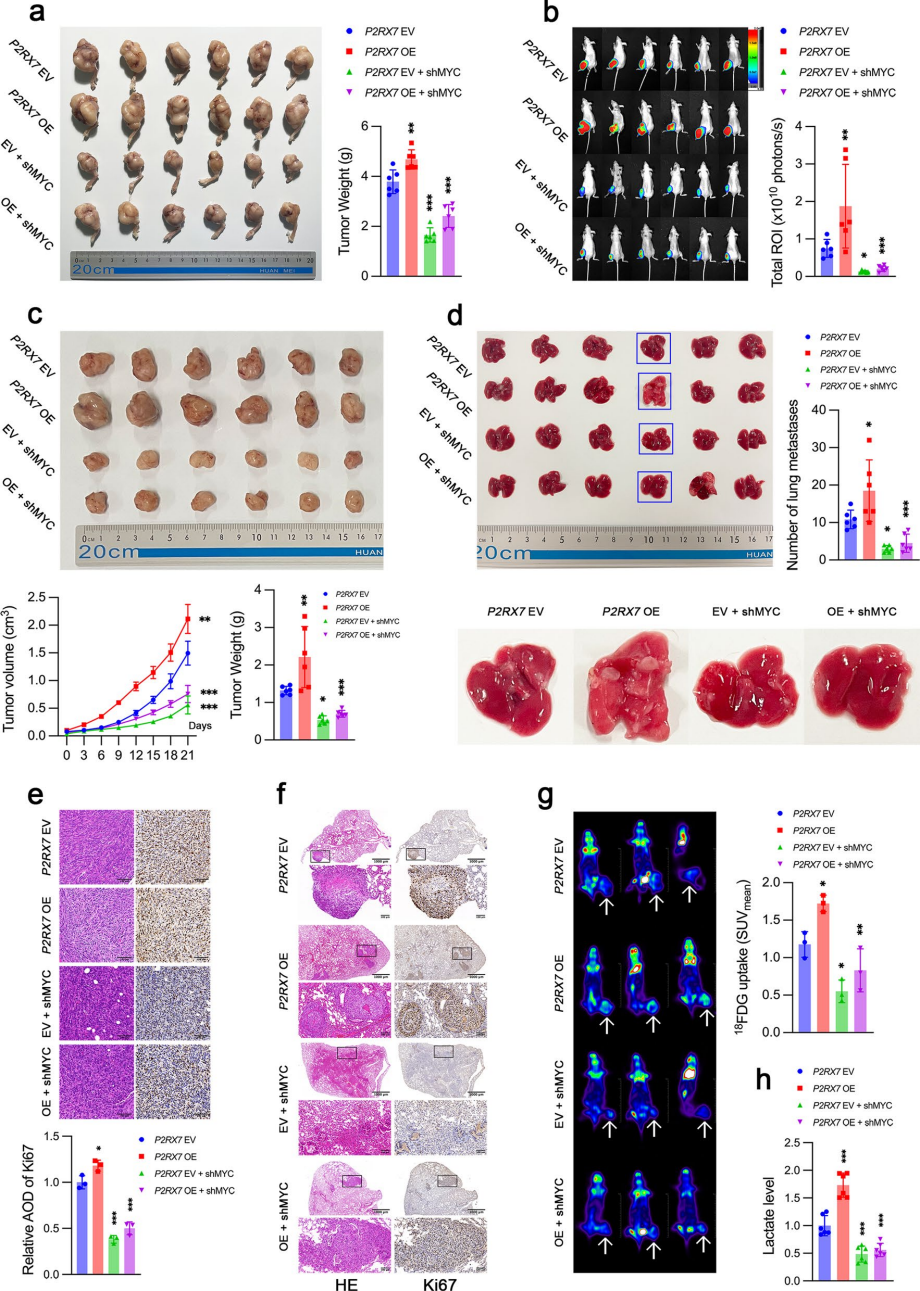
P2RX7 facilitated osteosarcoma growth and lung metastasis via c-Myc through metabolic reprogramming in vivo. **a** MNNG/HOS cells were transfected with empty vector (*P2RX7* EV), *P2RX7* plasmid (*P2RX7* OE), EV plus *MYC* shRNA (*P2RX7* EV + shMYC) and OE plus shMYC (*P2RX7* OE + shMYC) respectively and then injected into mouse tibia. Stripped orthotopic osteosarcoma in tibia were shown and tumor weight were measured. **b** Bioluminescence images of tumor-bearing mice from **a**. The total luminous intensity of the region of interest (ROI) was shown. **c** Subcutaneous tumor model was established by injecting tumor cells transfected as in **a**. Tumor volume was monitored every 3 days and growth curve was then plotted. Tumor were weighed after isolation. **d** Lung tissue was resected in mice from **a** and metastatic nodules were counted. **e** Representative images of histological analysis, including Hematoxylin and Eosin (HE) staining and immunohistochemistry (IHC) staining with Ki67 antibody. Average optical density (AOD) of IHC was calculated. **f** Histological analysis of HE and IHC for lung tissue with metastasis. **g** Representative ^18^F-FDG PET/CT images of mice from **a** (n = 3). Arrows indicated tumor glucose uptake. **h** Lactate level of tumor tissues was detected in mice from **a**. Data are shown as mean ± standard deviation (SD). *p < 0.05, **p < 0.01, ***p < 0.001 versus corresponding *P2RX7* EV or OE group

We established orthotopic and subcutaneous tumor models in tibia and axilla, respectively, to determine the role of c-Myc in P2RX7-induced tumor growth and metastasis. Obviously, c-Myc knockdown greatly impaired the ability of P2RX7 to facilitate tumor growth (Fig. 8a), which was further confirmed by bioluminescence examination (Fig. 8b). Consistent with in situ tumor model, downregulation of c-Myc dramatically attenuated the tumor growth-promoting effect of P2RX7 (Fig. 8c). It was noteworthy that P2RX7 overexpression markedly increased the number of lung metastases, which could be largely reduced by c-Myc knockdown (Fig. 8d). In addition, histological analysis indicated that P2RX7 overexpression significantly enhanced the intensity of Ki67 staining, whereas c-Myc knockdown obviously diminished such enhanced staining (Fig. 8e). In terms of lung tissue, Ki67 staining was much stronger at metastatic nodules than that at surrounding areas (Fig. 8f). Moreover, the overall intensity of Ki67 staining appeared to be higher in the P2RX7 overexpression group than that in the c-Myc knockdown group. More importantly, ^18^F-FDG PET/CT scan was carried out to evaluate glucose uptake in tumor xenografts in vivo. Tumors with P2RX7 overexpression cells presented increased glucose uptake, while c-Myc knockdown blocked the ability of P2RX7 to promote glucose uptake (Fig. 8g). Furthermore, we detected lactate level within tumor tissue and found that c-Myc knockdown almost abrogated P2RX7-stimulated lactate production (Fig. 8h). Thus, we concluded that P2RX7 promoted tumor growth and metastasis and metabolic reprogramming mainly dependent on c-Myc.

## Discussion

P2RX7 is a ligand-gated transmembrane channel that has attracted increasing attention in cancer. Upregulation and activation of P2RX7 have been reported in many malignancies and is considered as an oncogene, despite controversial studies. Expression of P2RX7 has been validated in several OS cell lines and exhibits various biological functions [50–52]. Moreover, P2RX7 deletion in mice reveals its role in the regulation of bone homeostasis [53]. In our previous study [40], we have determined that P2RX7 plays a critical role in OS progression. However, the potential mechanisms by which P2RX7 promotes tumor growth and metastasis are not fully elucidated. Unlike normal cells, cancer cells frequently display a high rate of glucose metabolism, a hall mark of cancer [30]. This metabolic reprogramming confers rapid tumor growth by providing sufficient energy for enhanced biosynthesis. The Warburg effect (aerobic glycolysis) has been widely recognized, whereby cancer cells prefer glycolysis to oxidative respiration for energy production even in the presence of abundant oxygen [54]. In fact, cancer metabolism is far mor complex than we currently anticipate. Cancer cells exhibit a high metabolic heterogeneity. Recently, there is growing evidence that cancer cells retain mitochondrial oxidative phosphorylation and even depend heavily on oxidative phosphorylation for rapid proliferation [13, 55]. As for OS, emerging research has provided evidence of metabolism dysregulation and potential mechanisms [56, 57]. For instance, AHA1 enhances metabolic activity via upregulating IDH1 and thus promotes OS progression [38]. The correlation between P2RX7 and cancer metabolic reprogramming has been discussed in a review [31].

In this study, we demonstrated that P2RX7 played a key role in metabolic reprogramming including enhanced glycolysis and oxidative phosphorylation in OS. Through constructing *P2RX7* knockout OS cell lines, we performed transcriptomics, metabolomics, and biofunctional analyses in vitro and in vivo. We found that P2RX7 could regulate the expression of many metabolism-related genes, as well as metabolites. GSEA and enrichment analyses suggested altered metabolic and proliferative pathways in response to *P2RX7* knockout. Consistently, *P2RX7* knockout significantly suppressed glucose consumption, ATP, pyruvate and lactate production, ECAR and OCR. In addition, both glycolytic inhibitor 2-DG and oxidative phosphorylation inhibitor oligomycin could abolish the ability of P2RX7 to promote cell proliferation and tumor growth. These findings demonstrated that P2RX7-mediated metabolic reprogramming is critical for OS aggressiveness. Next, we explored the underlying mechanisms by which P2RX7 modulated tumor metabolism. As a transcriptional factor, c-Myc is an essential proto-oncogene in several cancers and considered to be involved in diverse biological processes such as cell proliferation, cell cycle, invasion and metabolism [58–60]. Notably, a variety of metabolism-related genes have been identified to be regulated by c-Myc, which can reshape tumor metabolism toward a higher level and thereby meet the increased energy demands for rapid tumor growth [61]. Most recently, a series of articles have provided insights into the potential mechanisms by which c-Myc regulates tumor metabolism in multiple malignant tumors, including OS [36, 37, 47, 48]. Here, we indicated that P2RX7 increased c-Myc expression, facilitated nuclear transportation (coupled with S62 phosphorylation) and decreased ubiquitination-dependent degradation (coupled with T58 phosphorylation). Additionally, we also confirmed that metabolism reconstitution orchestrated by P2RX7 was largely dependent on c-Myc stabilization. Furthermore, c-Myc mediated metabolic reprogramming was crucial for P2RX7 to promote tumor growth and metastasis.

During tumorigenesis and progression, cell proliferation and aggressiveness require metabolic remodeling to support biosynthesis and provide energy. As an essential link and coordinator, c-Myc is not only involved in metabolic remodeling but also directly related to cell proliferation and invasion. *MYC* is a critical gene that regulates global gene expression, especially involved in cell proliferation, cell cycle and cell adhesion. *MYC* is estimated to affect about 15% of all genes and to be dysregulated in approximately 70% of tumors [62–64]. More than two decades ago, c-Myc was found to modulate cell cycle by targeting CDK4 [65]. Subsequently, an increasing number of studies have focused on the relationship between c-Myc and cell cycle. With the use of CDK4/6 inhibitors in preclinical and clinical settings, the relationship between c-Myc and CDK4/6 has been better elucidated. Importantly, c-Myc activation is responsible for both innate [66] and induced resistance [67, 68] to CDK4/6 inhibition therapy. Moreover, the combination of CDK4/6 inhibitors with CDK2 [69, 70] or ERK [71] inhibition can overcome c-Myc-dependent resistance. In addition, c-Myc and MMPs synergistically promote tumor metastasis [72]. Mechanistically, c-Myc upregulates MMPs expression in multiple ways, including activation of GP73 [73], miR-210 and lncRNA MIR210HG [74].

It is well documented that the interactions between tumor cells and tumor microenvironment (TME) are pivotal to the tumor fate. The composition of TME is complex, containing non-cancerous cells, extracellular matrix and specific physicochemical conditions. Extracellular ATP (eATP) within TME is well investigated as an important signaling molecule, which can regulate a series of biological processes by binding to P2RX7 [75]. It is notable that eATP concentration measured in tumors (50–500 μmol/L) are dramatically higher than those in healthy tissues (10–100 μmol/L) [76–78]. Such high eATP concentrations are compatible with the low affinity of P2RX7 to ATP. Tonic activation of P2RX7 by eATP will generate a cation-selective channel to support cell survival, whereas prolonged stimulation leads to non-selective macropore formation to induce cell apoptosis. Furthermore, P2RX7 tonic stimulation improves both glycolysis and oxidative phosphorylation efficiency [33, 79]. Conversely, its overstimulation results in mitochondrial damage and cell death [80]. The permeability of P2RX7 macropore to large molecules was originally thought to arise after the opening of ion channel and only after prolonged activation. Now, it is clearly recognized that macropore formation is intrinsic to P2RX7 and occurs simultaneously with channel opening [17]. Therefore, it is necessary to explore how tumor cells gain the survival advantage of P2RX7 while avoid the damage caused by P2RX7 macropore [81]. P2RX7 splice variants and single-nucleotide polymorphisms (SNPs) have been broadly identified, which display distinguish functions in pathogenesis. Among them, P2X7B and nfP2X7 isoforms have attracted the most attention due to their inability to form macropore followed by cell death [28, 82]. Preferential expression of P2RX7B variant has also been observed in OS, which regulates OS growth and metastasis [39]. Moreover, eATP induces nfP2RX7 expression and thus enhances tumor cell survival [28]. Another explanation is that MMP2-dependent cleavage of P2RX7 enables cancer cells to evade P2RX7-mediated cytotoxicity [83]. Besides, the special TME conditions like hypoxia and acidosis may also have influence on P2RX7 function and expression [30, 84, 85].

On one hand, high concentration of eATP acts on P2RX7 to initiate various biological processes. On the other hand, P2RX7 contributes to the increase of eATP concentration by facilitating ATP generation and release. Activation of P2RX7 can enhance glucose metabolism including both glycolysis and oxidative respiration and thus promote intracellular ATP production. In response to multiple stimuli like hypoxia and plasma membrane damage, intracellular ATP can be released into TME in both passive and active ways, resulting in elevation of eATP concentration [81, 86]. P2RX7 is also involved in the process of intracellular ATP release [87–89]. Rapid tumor growth is in part dependent on P2RX7 stimulation through autocrine/paracrine eATP. P2RX7 activation by high concentration of eATP may in turn further promote ATP production and release, thus creating positive feedback. Biosynthesis and metabolism are maintained at a high level in tumor cells, which is partially attributed to the key regulator P2RX7. Reasonably, breaking this vicious circle by inhibiting P2RX7 might shed light on the development of novel tumor therapy.

As mentioned above, typical features of TME like hypoxia and acidosis can modulate cancer metabolism as well as P2RX7 function [90–92]. Thus, we further examined whether P2RX7 still maintained its function to regulate metabolism and cellular behavior under hypoxia in OS. As expected, P2RX7 facilitated glucose metabolism and promoted cell proliferation and aggression under hypoxia. Consistent with previous reports, our results showed that P2RX7 also enhanced cell survival and growth in serum starvation [33, 93]. A correlation between metabolic reprogramming and therapy resistance has already been described in cancer [94]. Moreover, administration of metabolic modifiers such as 2-DG and rotenone exhibits encouraging efficiency to overcome therapy resistance [95, 96]. Here, we demonstrated that P2RX7 enhanced the resistance of OS cells to CDDP, partially via c-Myc. P2RX7 inhibition might obtain exciting effects not only on suppression of glucose metabolism but also on improvement of chemotherapy sensitivity. Although the mechanisms by which P2RX7 affects therapeutic resistance through metabolic remodeling need to be further elucidated.

## Conclusions

Our study confirms the promoting effect of P2RX7 in OS progression and provides new insights into the role of P2RX7 in metabolic reprogramming. We further elucidate that P2RX7 enhances glucose metabolism and tumor growth and metastasis by increasing c-Myc stabilization. Mechanistically, P2RX7 facilitates nuclear transport of pS62 c-Myc while reduces the export of pT58 c-Myc to cytoplasm, where it undergoes ubiquitination-dependent degradation. These findings provide new evidence that P2RX7 might be a promising diagnostic and/or therapeutic target in OS. Furthermore, novel therapeutic strategies targeting metabolic reprogramming may lead to a breakthrough in OS treatment and ultimately improve patient prognosis.

## Supplementary Information

**Additional file 1: Table S1.** The sequences of primers used in this study.

**Additional file 2: Figure S1.** The effect of P2RX7 on cell biological functions by Gene Set Enrichment Analysis (GSEA) and pathway enrichment analyses. **a**, **b** GSEA analyses implied that P2RX7 could be a positive regulator of cell proliferation via accelerating cell cycle transition (**a**) and promoting DNA replication (**b**). **c** GSEA analyses suggested that P2RX7 was associated with ubiquitination-mediated protein degradation. **d** Kyoto Encyclopedia of Genes and Genomes (KEGG) pathway enrichment analyses of RNA-seq data in MNNG/HOS cells. **e** Gene Ontology (GO) pathway enrichment analyses of RNA-seq data in MNNG/HOS cells. **f** Oxidative phosphorylation was included in the top 20 of KEGG enriched pathways based on metabolomics in MNNG/HOS cells.

**Additional file 3: Figure S2.** P2RX7 improved chemoresistance of osteosarcoma cells via c-Myc. **a** Cell sensitivity to cisplatin (CDDP) was reduced in *P2RX7* knockout (KO) MNNG/HOS cells compared to *P2RX7* wild-type (WT) cells. **b** P2RX7 suppressed cell apoptosis by flowcytometry analysis. **c** MNNG/HOS cells were transfected with empty vector (*P2RX7* EV), *P2RX7* plasmid (*P2RX7* OE), EV plus *MYC* shRNA (*P2RX7* EV + shMYC) and OE plus shMYC (*P2RX7* OE + shMYC) respectively and cell apoptosis was analyzed by flowcytometry. Data are shown as mean ± standard deviation (SD). *p < 0.05, **p < 0.01, ***p < 0.001 versus corresponding *P2RX7* EV or OE group or as indicated.

**Additional file 4: Figure S3.** The role of P2RX7 on cell biological behaviors under hypoxia and serum starvation. **a**, **f** Cell proliferation was facilitated in *P2RX7* wild-type (WT) MNNG/HOS cells compared to *P2RX7* knockout (KO) cells under hypoxia. **b**, **g** P2RX7 facilitated cell migration in osteosarcoma cells. **c**, **h** Cell invasiveness was increased in *P2RX7* WT cells under hypoxia. **d**, **i** The promoting effects of P2RX7 on glucose consumption, glucose uptake, lactate and ATP production under condition of hypoxia. **e, j** P2RX7 increased cell survival in serum starvation. Data are shown as mean ± standard deviation (SD). *p < 0.05, **p < 0.01, ***p < 0.001 versus corresponding *P2RX7* WT group.

**Additional file 5: Figure S4.** P2RX7 regulated cell proliferation and tumor growth via glucose metabolism. **a** Both glucose metabolism inhibitors 2-DG (2.5 mM) and oligomycin (100 μM) attenuated cell proliferation. **b** MNNG/HOS cells were transfected with empty vector (*P2RX7* EV) and *P2RX7* plasmid (*P2RX7* OE) and 2-DG (2.5 mM) and oligomycin (100 μM) were used. Then, cell proliferation assay was performed. **c** Inhibition of glucose metabolism suppressed tumor growth in *vivo*. Data are shown as mean ± standard deviation (SD). *p < 0.05, **p < 0.01, ***p < 0.001 versus control (Con) group or corresponding *P2RX7* EV or OE group.

**Additional file 6: Figure S5.** P2RX7 regulated expression of glucose metabolism related genes through c-Myc. **a**, **c** Osteosarcoma cells were transfected with empty vector (*P2RX7* EV), *P2RX7* plasmid (*P2RX7* OE), EV plus *MYC* shRNA (*P2RX7* EV + shMYC) and OE plus shMYC (*P2RX7* OE + shMYC) respectively. Glycolysis-related gene expression was detected by qRT-PCR. **b**, **d** Oxidative phosphorylation-related gene expression was analyzed by qRT-PCR in *P2RX7* EV, *P2RX7* OE, *P2RX7* EV + shMYC and *P2RX7* OE + shMYC groups. Data are shown as mean ± standard deviation (SD). *p < 0.05, **p < 0.01, ***p < 0.001 versus corresponding *P2RX7* EV or OE group.

## Data Availability

All data generated or analyzed during this study are included in this published article and its supplementary information files. The datasets used and/or analyzed during the current study are available from the author (GS, email: gaohongsheng@hust.edu.cn) on reasonable request.

## Abbreviations

OS: Osteosarcoma
SIX1: Transcription factor sine oculis homeobox 1
P2RX7: Purinergic receptor P2X 7
TME: Tumor microenvironment
eATP: Extracellular Adenosine triphosphate
DMEM/F12: Dulbecco’s modified Eagle’s medium F12
FBS: Fetal bovine serum
sgRNA: Single guide RNA
CCK-8: Cell Counting Kit-8 assay
OD: Optical density
PBS: Phosphate buffered saline
KRPH: Krebs–Ringer-Phosphate-HEPES
LDH: Lactate dehydrogenase
ECAR: Extracellular acidification rate
OCR: Oxygen consumption rate
FCCP: P-trifluoromethoxy carbonyl cyanide phenylhydrazone
Rote/AA: Rotenone plus antimycin A
PFKM: Fragments per kilobase of exon model per million mapped fragments
GO: Gene Ontology
KEGG: Kyoto Encyclopedia of Genes and Genomes
GSEA: Gene Set Enrichment Analysis
HPLC–MS/MS: High performance liquid chromatography-tandem mass spectrometric
RT-PCR: Reverse transcription-polymerase chain reaction
RIPA: Radio immunoprecipitation assay lysis buffer
BCA: Bicinchoninic Acid Assay
BSA: Bovine serum albumin
TBST: Tris-buffered Saline-Tween solution
HRP: Horse-radish peroxidase
ECL: Enhanced chemiluminescence
PET/CT: Positron emission tomography/computed tomography
SUV: Standard uptake value
HE: Hematoxylin and eosin
IHC: Immunohistochemistry
DAB: 3, 3-Diaminobenzidiine tetrahydrochloride
SD: Standard deviation
ANOVA: One-way analysis of variance
KO: Knockout
WT: Wild type
SLC2A1: Solute carrier family 2 member 1
SLC2A4: Solute carrier family 2 member 4
HK1: Hexokinase 1
HK2: Hexokinase 2
GPI: Glucose-6-phosphate isomerase
PFKL: Phosphofructokinase, liver type
PFKP: Phosphofructokinase, platelet
PFKM: Phosphofructokinase, muscle
TPI1: Triosephosphate isomerase 1
GAPDH: Glyceraldehyde-3-phosphate dehydrogenase
PGK1: Phosphoglycerate kinase 1
PGAM1: Phosphoglycerate mutase 1
ENO1: Enolase 1
PKM: Pyruvate kinase M
ALDOA: Aldolase A
ALDOB: Aldolase B
ALDOC: Aldolase C
LDHA: Lactate dehydrogenase A
LDHB: Lactate dehydrogenase B
PDHA1: Pyruvate dehydrogenase E1 subunit alpha 1
PDHB: Pyruvate dehydrogenase E1 subunit beta
DLAT: Dihydrolipoamide S-acetyltransferase
DLD: Dihydrolipoamide dehydrogenase
CS: Citrate synthase
ACO1: Aconitase 1
ACO2: Aconitase 2
IDH1: Isocitrate dehydrogenase (NADP( +)) 1
IDH2: Isocitrate dehydrogenase (NADP( +)) 2
IDH3A: Isocitrate dehydrogenase (NADP( +)) 3 catalytic subunit alpha
OGDH: Oxoglutarate dehydrogenase
SDHA: Succinate dehydrogenase complex flavoprotein subunit A
SDHB: Succinate dehydrogenase complex flavoprotein subunit B
FH: Fumarate hydratase
MDH2: Malate dehydrogenase 2
SUCLG1: Succinate-CoA ligase GDP/ADP-forming subunit alpha
PCNA: Proliferating cell nuclear antigen
CDK4: Cyclin dependent kinase 4
MMP2: Matrix metallopeptidase 2
MMP9: Matrix metallopeptidase 2
IC50: Half maximal inhibitory concentration
CDDP: Cisplatin
SNPs: Single-nucleotide polymorphisms
