# Supplementary material for: P2RX7 promotes osteosarcoma progression and glucose metabolism by enhancing c-Myc stabilization
Source: J Transl Med. 2023 Feb 20;21:132. doi: 10.1186/s12967-023-03985-z (PMC9940387; doi:10.1186/s12967-023-03985-z)
Supplement: Supplementary file 1 — Additional file 1: Table S1. The sequences of primers used in this study. [file 12967_2023_3985_MOESM1_ESM.docx]

Table S1. The sequences of primers used in this study.

| ***Gene*** | **Forward (5’-3’)** | **Reverse (5’-3’)** |
| --- | --- | --- |
| *TUBA1* | GGAACCCACAGTCATTGATGAAG | CGAGGTGAACCCAGAACCAG |
| *GLUT1* | TGGCATCAACGCTGTCTTCT | CTAGCGCGATGGTCATGAGT |
| *GLUT4* | GTCGGGCTTCCAACAGATAGG | AGCTCTGTTCAATCACCTTCTGAG |
| *HK1* | TGGACTCATTGTTGGGACCG | TCAGAGATCTGCCCTCGGAA |
| *HK2* | AACACCGGTCGCTTTGAGAC | GTAGACGCTTGGCAAAATGGG |
| *GPI* | CAACTACACCGAGGGTCGAG | CGGGGACCTCCTGAAGAGTA |
| *PFKL* | TCGACACCCGTGTAACTGTG | TTGTTCTCGAAGCTCCCACC |
| *PFKM* | GCTGACACCTTCCGTTCTGA | GACAAGGGCCAGGTATCCAC |
| *PFKP* | GGACTGGGTGTTCCTTCCAG | GCGTGACGACAAGCTCTTTG |
| *ALDOA* | CAAATCCAAGGGCGGTGTTG | ACAATGCCATTCTGCTGGC |
| *ALDOB* | AAGAAACCACCATTCAAGGGCT | GATAGCGAGGCTGGATGGAC |
| *ALDOC* | CTCAATGCCATCAACCGCTG | CTGCAAGCCCATTCACCTCA |
| *TPI1* | GGACTCGGAGTAATCGCCTG | GTACTTCCTGGGCCTGTTGG |
| *GAPDH* | TGCACCACCAACTGCTTAGC | GGCATGGACTGTGGTCATGAG |
| *PGK1* | TGTAGGCCCAGAAGTGGAGA | CTGGCTCGGCTTTAACCTTG |
| *PGAM1* | AGCATCTGGAGGGTCTCTCT | ATCCCCCAGAAACTGCATGG |
| *ENO1* | GGAGATCTCGCCGGCTTTAC | CAGCAGCTCTGAAGAGACCTTT |
| *PKM* | GAATCATGAGGGGGTTCGGA | CATGCTCTCCAGCATCTGAGTA |
| *LDHA* | TCTTGACCTACGTGGCTTGG | CTCCATACAGGCACACTGGAA |
| *LDHB* | GGGAGAGTCGGCTCAATCTG | ACTCCACACAGCCACACTTG |
| *PDHA1* | ATTCCTGGGCTGAGAGTGGA | CCACACTGGCAAGATTGCTG |
| *PDHB* | TGGGGCATACAAGGTTAGTCG | ATTCACAAATGGGCCGCAAC |
| *DLAT* | AACTCCCCAGCCTTTAGCTC | GCTGCCGGAGCAGGAG |
| *DLD* | CCCAGCGGAGGTGAAAGTAT | TTGAAATGGCCTCTCTTGGC |
| *CS* | GGGGTGCTGCTCCAGTATTA | CTAAGGCTCGGCTCCAGATG |
| *ACO1* | CTGCAGGACTTTACGGGTGT | GCAGGGCAGACAGGGTTTAT |
| *ACO2* | CAGTACCATGTGGCCTCAGTC | CGGCCGGTTCAGTCGTTT |
| *IDH1* | CTCTGTGGCCCAAGGGTATG | GGATTGGTGGACGTCTCCTG |
| *IDH2* | ACAACACCGACGAGTCCATC | AAGCCACCCGAAGACTTGAG |
| *IDH3A* | GCGTGGATCTCTAAGGTCTCTC | TCTGGGCCAATACCATCTCCT |
| *OGDH* | GAATGGCGTGGACTACGTGA | CACATCTCCGGAGCCCTCAT |
| *SUCLG1* | CTTTGTGCGTTGGCATTGGA | TTCTCCCAGGAGGAGCAGTT |
| *SDHA* | CAGTTCCACCCTACAGGCAT | GGGGCGTATCGCTCCATAAA |
| *SDHB* | ACTCTAGCTTGCACCCGAAG | TCGTAGAGCCCGTCCAGTTT |
| *FH* | TGTTGCTGTCACTGTCGGAG | TTTGCTGCCTTGTCATACCCTAT |
| *MDH2* | CTGCTGCTTGGGAAAAAGGG | CAACGGCTTTGCAGTGACAT |
| *MYC* | TTCCCCTACCCTCTCAACGA | ATCTTCTTGTTCCTCCTCAGAGT |
| *MMP2* | CGACCACAGCCAACTACGAT | GTCAGGAGAGGCCCCATAGA |
| *MMP9* | GCCTCTGGAGGTTCGACG | CTGGTTCAACTCACTCCGGG |
| *PCNA* | TCCTGTGCAAAAGACGGAGT | CATCCTCGATCTTGGGAGCC |
| *CDK4* | CAATTGCATCGTTCACCGAGA | GGCAGCCCAATCAGGTCAAAG |
