# Supplementary figures and images for: P2RX7 promotes osteosarcoma progression and glucose metabolism by enhancing c-Myc stabilization
Source: J Transl Med. 2023 Feb 20;21:132. doi: 10.1186/s12967-023-03985-z (PMC9940387; doi:10.1186/s12967-023-03985-z)

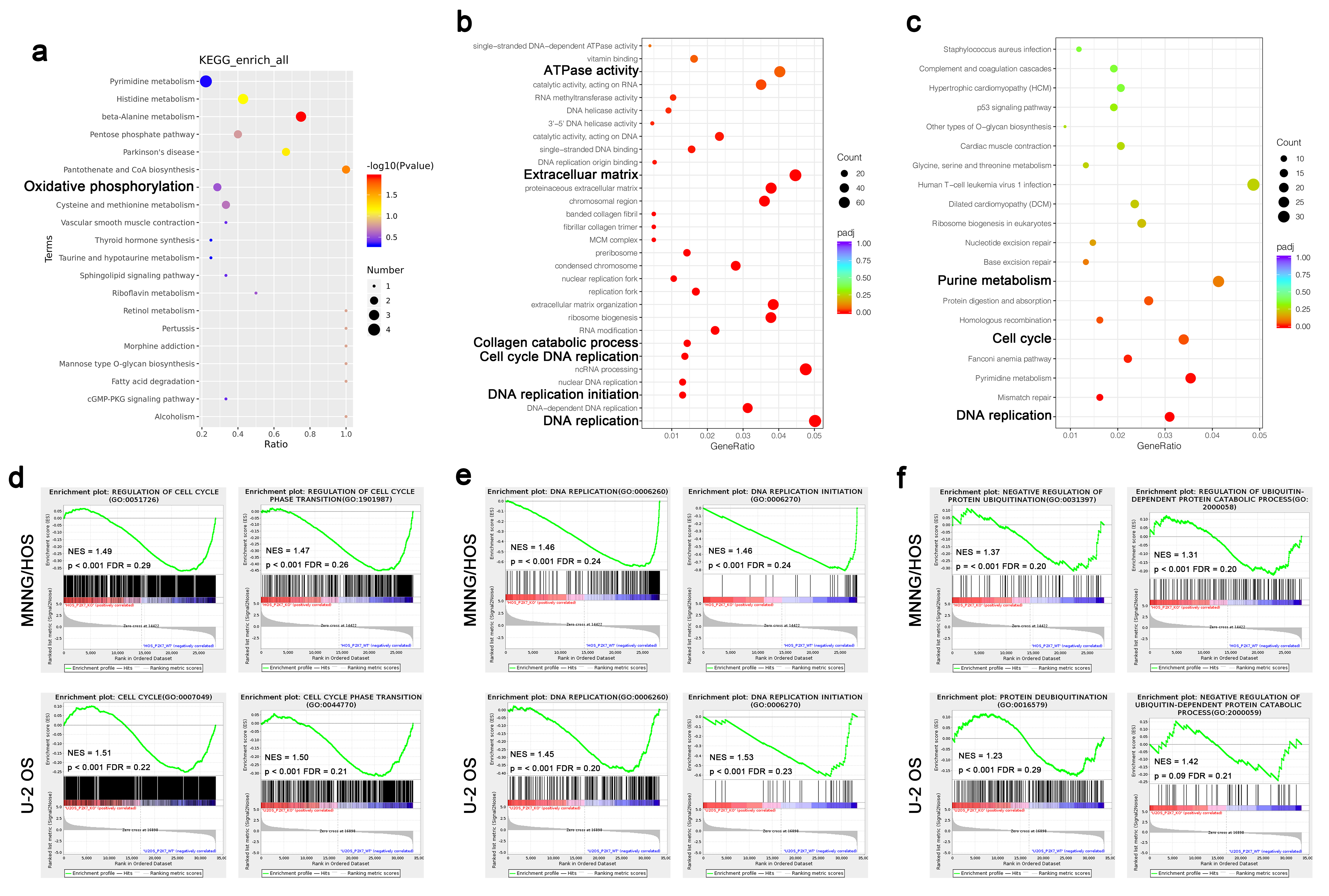

Supplement: Supplementary file 2 — Additional file 2: Figure S1. The effect of P2RX7 on cell biological functions by Gene Set Enrichment Analysis (GSEA) and pathway enrichment analyses. a, b GSEA analyses implied that P2RX7 could be a positive regulator of cell proliferation via accelerating cell cycle transition (a) and promoting DNA replication (b). c GSEA analyses suggested that P2RX7 was associated with ubiquitination-mediated protein degradation. d Kyoto Encyclopedia of Genes and Genomes (KEGG) pathway enrichment analyses of RNA-seq data in MNNG/HOS cells. e Gene Ontology (GO) pathway enrichment analyses of RNA-seq data in MNNG/HOS cells. f Oxidative phosphorylation was included in the top 20 of KEGG enriched pathways based on metabolomics in MNNG/HOS cells. [file 12967_2023_3985_MOESM2_ESM.tif]

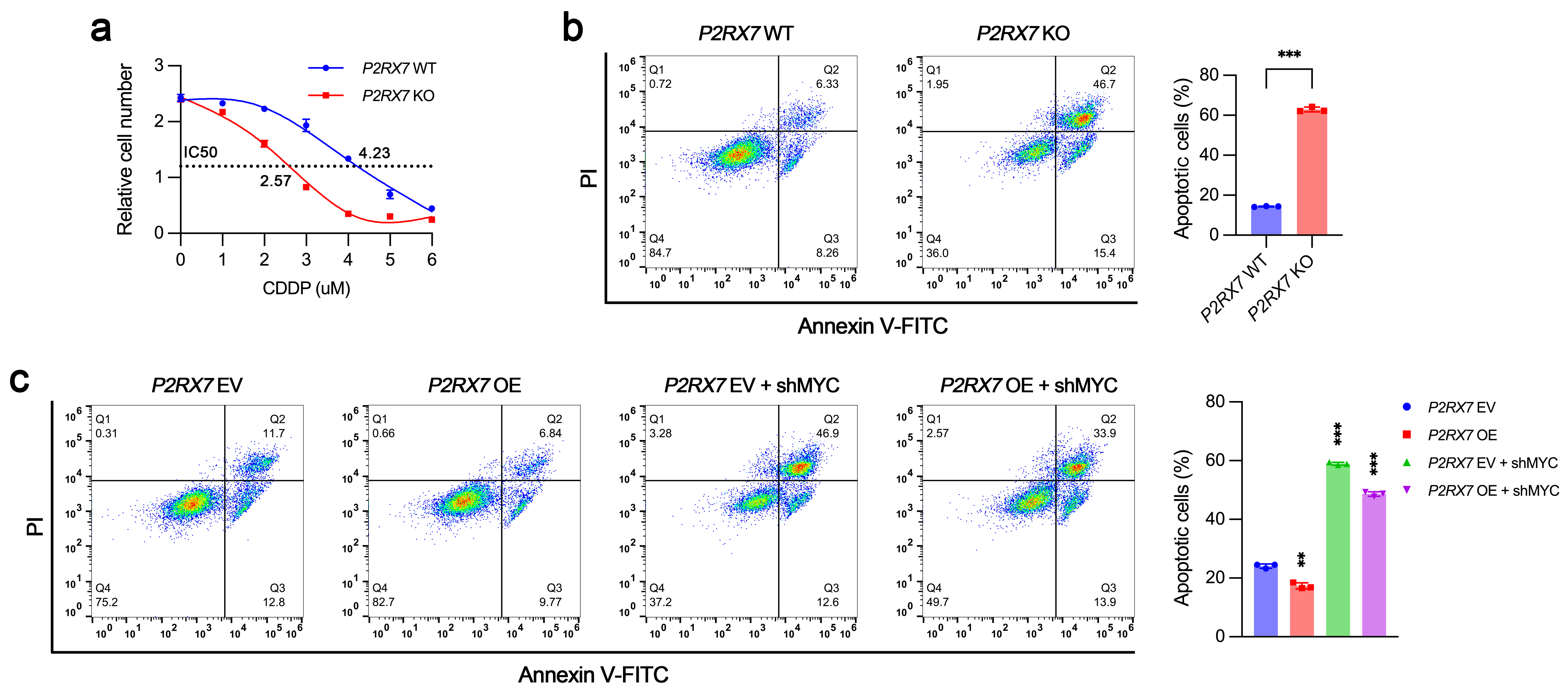

Supplement: Supplementary file 3 — Additional file 3: Figure S2. P2RX7 improved chemoresistance of osteosarcoma cells via c-Myc. a Cell sensitivity to cisplatin (CDDP) was reduced in P2RX7 knockout (KO) MNNG/HOS cells compared to P2RX7 wild-type (WT) cells. b P2RX7 suppressed cell apoptosis by flowcytometry analysis. c MNNG/HOS cells were transfected with empty vector (P2RX7 EV), P2RX7 plasmid (P2RX7 OE), EV plus MYC shRNA (P2RX7 EV + shMYC) and OE plus shMYC (P2RX7 OE + shMYC) respectively and cell apoptosis was analyzed by flowcytometry. Data are shown as mean ± standard deviation (SD). *p < 0.05, **p < 0.01, ***p < 0.001 versus corresponding P2RX7 EV or OE group or as indicated. [file 12967_2023_3985_MOESM3_ESM.tif]

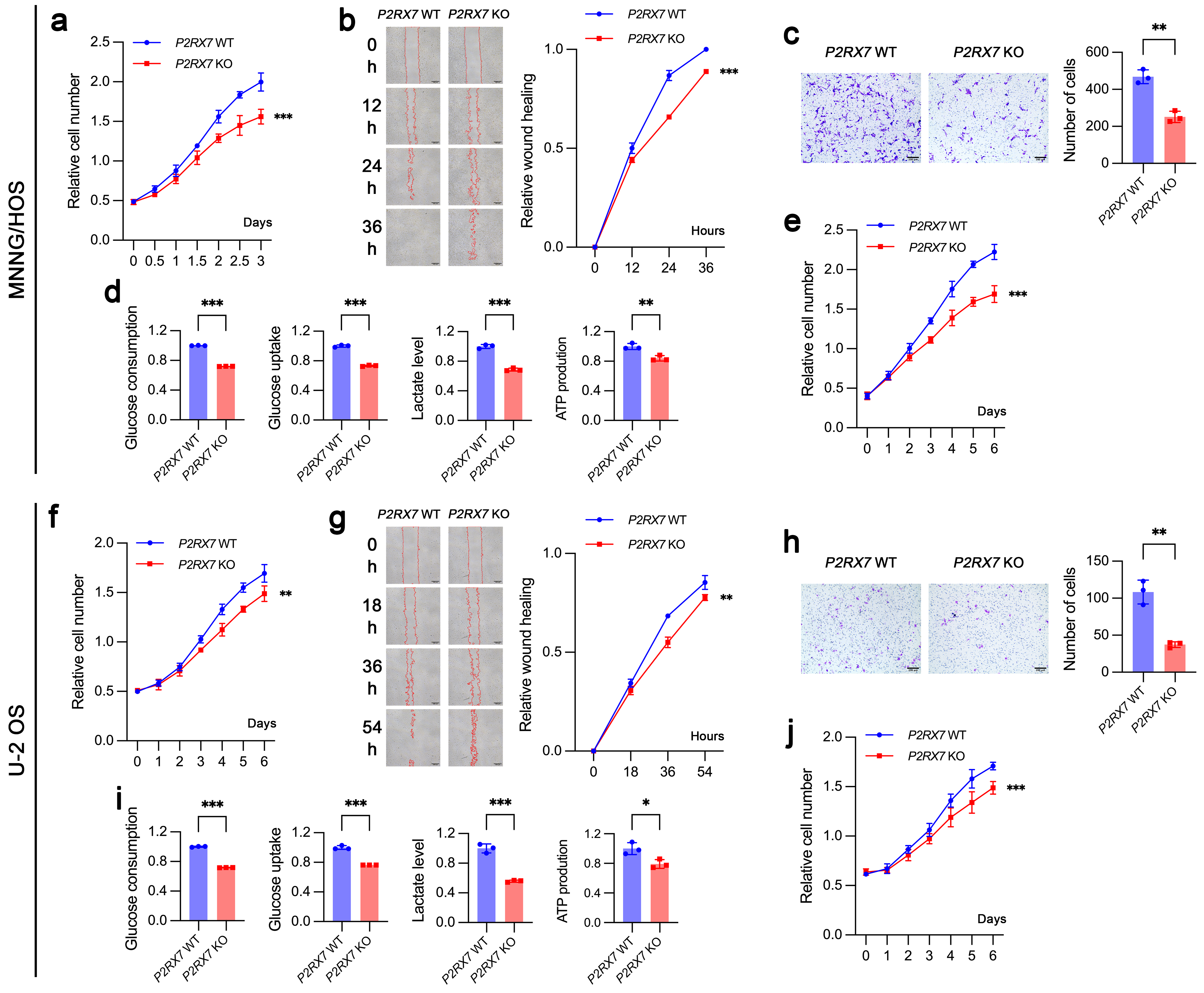

Supplement: Supplementary file 4 — Additional file 4: Figure S3. The role of P2RX7 on cell biological behaviors under hypoxia and serum starvation. a, f Cell proliferation was facilitated in P2RX7 wild-type (WT) MNNG/HOS cells compared to P2RX7 knockout (KO) cells under hypoxia. b, g P2RX7 facilitated cell migration in osteosarcoma cells. c, h Cell invasiveness was increased in P2RX7 WT cells under hypoxia. d, i The promoting effects of P2RX7 on glucose consumption, glucose uptake, lactate and ATP production under condition of hypoxia. e, j P2RX7 increased cell survival in serum starvation. Data are shown as mean ± standard deviation (SD). *p < 0.05, **p < 0.01, ***p < 0.001 versus corresponding P2RX7 WT group. [file 12967_2023_3985_MOESM4_ESM.tif]

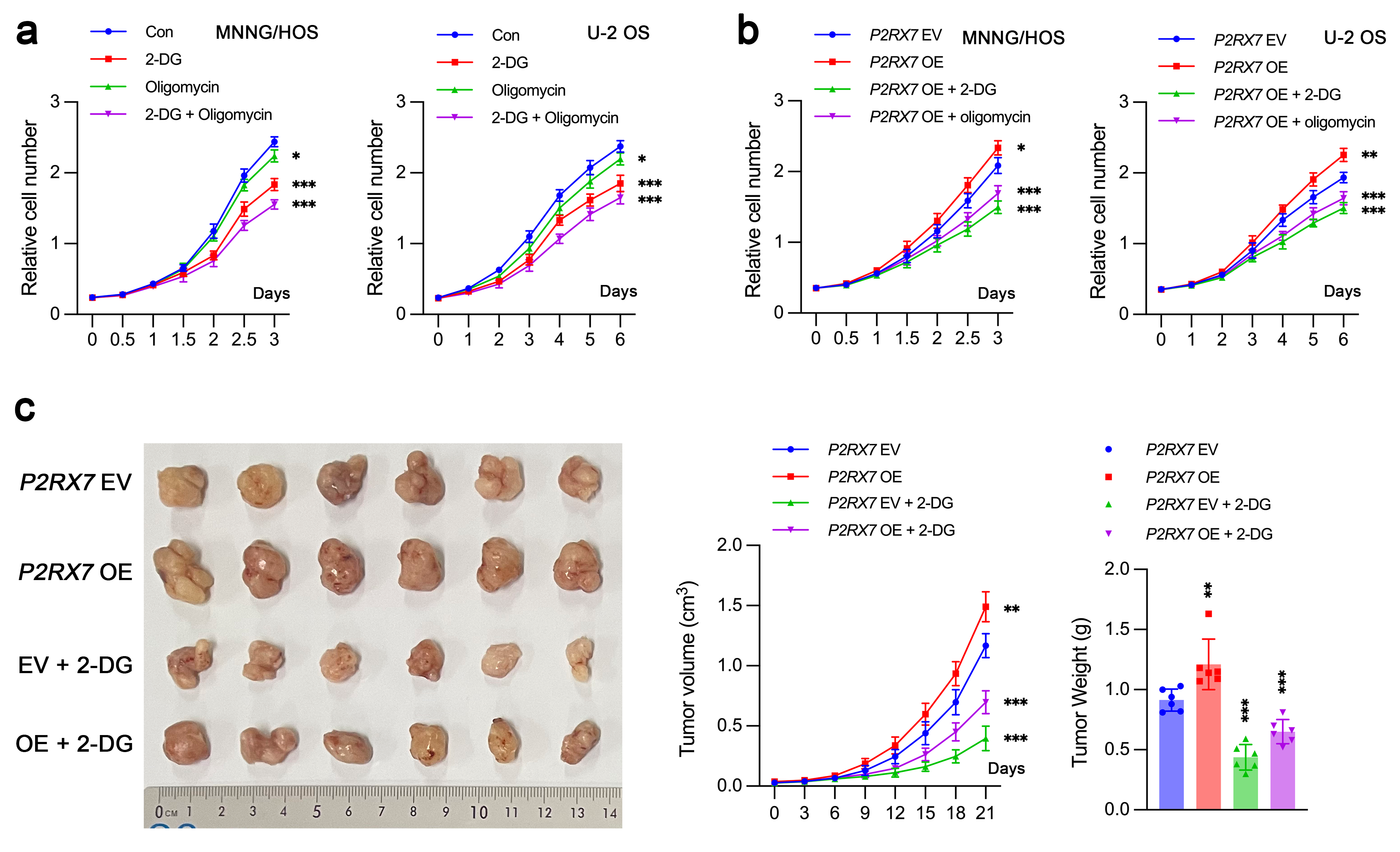

Supplement: Supplementary file 5 — Additional file 5: Figure S4. P2RX7 regulated cell proliferation and tumor growth via glucose metabolism. a Both glucose metabolism inhibitors 2-DG (2.5 mM) and oligomycin (100 μM) attenuated cell proliferation. b MNNG/HOS cells were transfected with empty vector (P2RX7 EV) and P2RX7 plasmid (P2RX7 OE) and 2-DG (2.5 mM) and oligomycin (100 μM) were used. Then, cell proliferation assay was performed. c Inhibition of glucose metabolism suppressed tumor growth in vivo. Data are shown as mean ± standard deviation (SD). *p < 0.05, **p < 0.01, ***p < 0.001 versus control (Con) group or corresponding P2RX7 EV or OE group. [file 12967_2023_3985_MOESM5_ESM.tif]

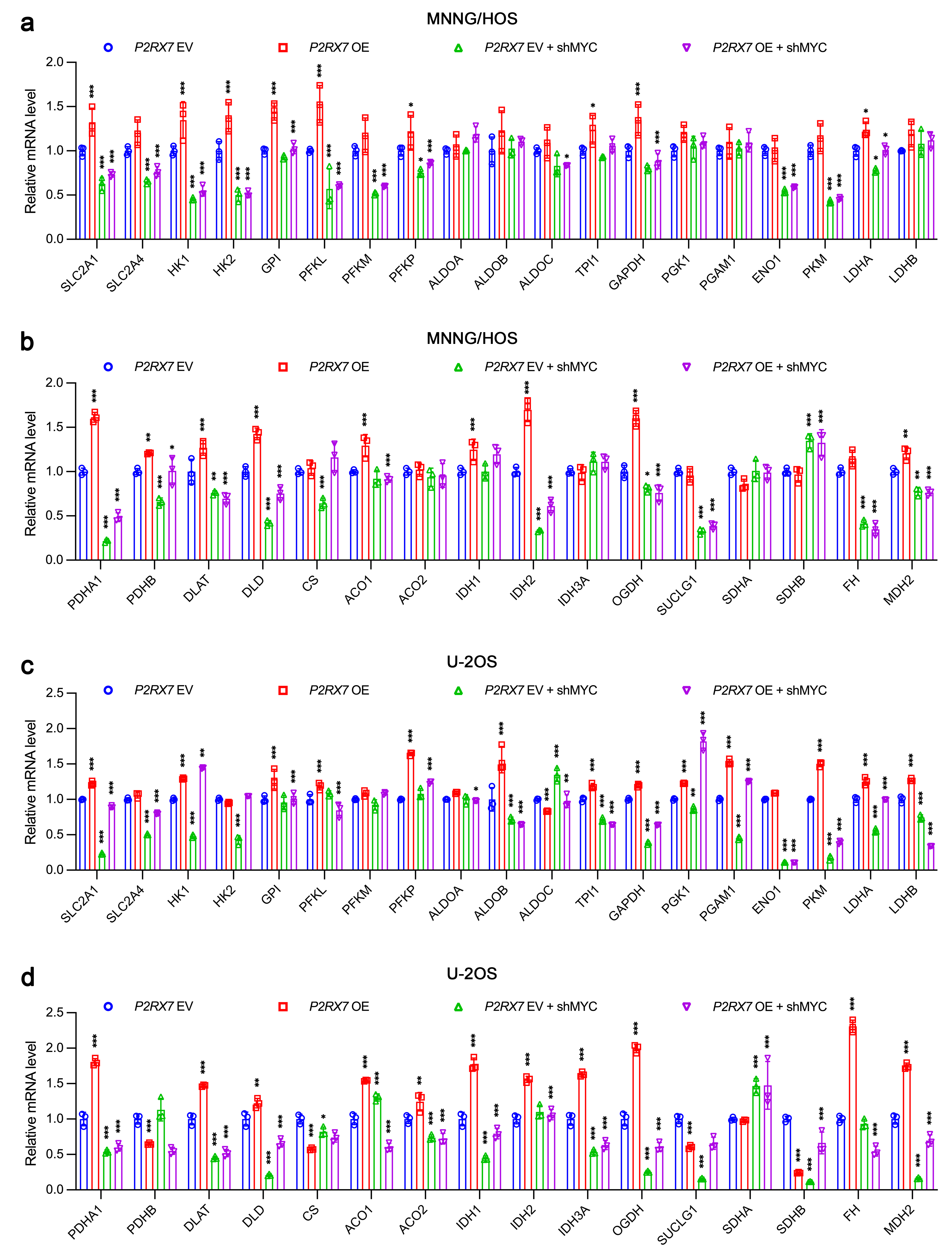

Supplement: Supplementary file 6 — Additional file 6: Figure S5. P2RX7 regulated expression of glucose metabolism related genes through c-Myc. a, c Osteosarcoma cells were transfected with empty vector (P2RX7 EV), P2RX7 plasmid (P2RX7 OE), EV plus MYC shRNA (P2RX7 EV + shMYC) and OE plus shMYC (P2RX7 OE + shMYC) respectively. Glycolysis-related gene expression was detected by qRT-PCR. b, d Oxidative phosphorylation-related gene expression was analyzed by qRT-PCR in P2RX7 EV, P2RX7 OE, P2RX7 EV + shMYC and P2RX7 OE + shMYC groups. Data are shown as mean ± standard deviation (SD). *p < 0.05, **p < 0.01, ***p < 0.001 versus corresponding P2RX7 EV or OE group. [file 12967_2023_3985_MOESM6_ESM.tif]
